# Jasmonate‐Activated AaWRKY9–AabHLH93/AabHLH93–AaMYB7 Complexes Balance Artemisinin Biosynthesis in *Artemisia annua*

**DOI:** 10.1111/pbi.70416

**Published:** 2025-10-28

**Authors:** Xueqing Fu, Han Zheng, Yaojie Zhang, Muyao Yu, Shu Li, Hang Liu, Pin Liu, Ling Li, Xiaofen Sun, Jingya Zhao, Yuliang Wang, Kexuan Tang

**Affiliations:** ^1^ School of Design Shanghai Jiao Tong University Shanghai China; ^2^ Plant Biotechnology Research Center, Fudan‐SJTU‐Nottingham Plant Biotechnology R&D Center, School of Agriculture and Biology Shanghai Jiao Tong University Shanghai China; ^3^ State Key Laboratory for Quality Ensurance and Sustainable Use of Dao‐di Herbs, National Resource Center for Chinese Materia Medica China Academy of Chinese Medical Sciences Beijing China; ^4^ School of Chinese Materia Medica Beijing University of Chinese Medicine Beijing China; ^5^ Shanghai Jiao Tong University Sichuan Research Institute Chengdu China

**Keywords:** *Artemisia annua*, artemisinin, jasmonate, spatiotemporal regulatory, transcription factor

## Abstract

Artemisinin, an antimalarial sesquiterpene lactone, is biosynthesized in glandular trichomes of *Artemisia annua* . Although numerous transcription factors have been demonstrated to regulate artemisinin biosynthesis, the molecular mechanisms underlying the high expression of artemisinin‐associated biosynthetic enzyme and transcription factor genes in young leaves and their marked decline during leaf maturation remain elusive. Here, we identify a trichome‐enriched bHLH transcription factor, AabHLH93, through yeast two‐hybrid screening using AaWRKY9 as bait. Yeast one‐hybrid and electrophoretic mobility shift assays demonstrate that AabHLH93 directly binds to the *AaCYP71AV1* promoter. Furthermore, overexpressing *AabHLH93* elevates artemisinin levels, while its RNAi suppresses artemisinin biosynthesis. In young leaves, elevated JA represses *AaMYB7* expression and triggers 26S proteasome‐mediated degradation of AaJAZ9. AabHLH93 physically interacts with AaWRKY9 through its C‐terminal domain to form the AaWRKY9–AabHLH93 complex, enhancing the activation of artemisinin biosynthetic genes. Conversely, age‐dependent accumulation of the R2R3‐MYB repressor AaMYB7 in mature trichomes disrupts this synergy. In mature leaves, age‐dependent JA depletion permits AaMYB7 upregulation and AaJAZ9 accumulation. AaMYB7 directly binds to AabHLH93's C‐terminal domain, displacing AaWRKY9 and hindering the formation of the AaWRKY9–AabHLH93 activation complex. These findings elucidate how antagonistic AabHLH93–AaWRKY9 and AabHLH93–AaMYB7 modules, coupled with JA and developmental cues, dynamically control artemisinin biosynthesis.

## 1 Introduction

Malaria remains a highly critical global health issue, with an alarming prevalence of approximately 247 million cases reported in 84 malaria‐endemic countries. This staggering figure reflects a concerning rise of 2 million cases when compared to the previous year, indicating a worrisome trend in malaria incidence. Artemisinin combination therapies (ACT) are still recommended as the preferred drug to fight malaria. Artemisinin is a compound known as a sesquiterpene lactone, which is derived from the plant *Artemisia annua* . It has gained recognition for its widespread application in the treatment of malaria (Greenwood and Mutabingwa 2002). Besides, artemisinin and its semisynthetic derivative have demonstrated inhibitory effects on various viruses (Thomas et al. 2008), shown potent anticancer activity (Crespo‐Ortiz and Wei 2012), and unique anti‐inflammatory properties (Wang et al. 2007). In *A. annua* , artemisinin is synthesized within the glandular trichomes, which consist of two stalk cells, two basal cells and three pairs of secretory cells (Duke et al. 1994; Olsson et al. 2009). Significant endeavours have been undertaken to elucidate the biosynthetic pathway of artemisinin (Shen et al. 2018). The biosynthesis of artemisinic acid (AA) and dihydroartemisinic acid (DHAA) encompasses the action of multiple enzymes, such as amorpha‐4,11‐diene synthase (ADS), cytochrome P450 monooxygenase (CYP71AV1), cytochrome P450 reductase (CPR), artemisinic aldehyde D‐11(13)‐double bond reductase (DBR2), an aldehyde dehydrogenase (ALDH1), an alcohol dehydrogenase (ADH1) and cytochrome b5 monooxygenase (CYB5) (Bouwmeester et al. 1999; Chang et al. 2000; Paddon et al. 2013; Teoh et al. 2009; Zhang et al. 2008). Moreover, the transformations of DHAA into artemisinin and AA into arteannuin B take place via nonenzymatic photo‐oxidation reactions (Brown and Sy 2004, 2007).

Although the biosynthetic pathway of artemisinin has been well characterised, the mechanisms underlying the transcriptional regulation of the genes within this pathway remain mostly unknown. Research has highlighted that various transcription factors (TFs) are involved in the regulation of artemisinin biosynthesis in *A. annua* . AaWRKY1, for instance, was shown to boost artemisinin levels by activating the expression of *AaADS*, *AaCYP71AV1* and *AaDBR2* in AaWRKY1‐overexpressing lines (Ma et al. 2009). Furthermore, several APETALA 2/ETHYLENE RESPONSIVE FACTOR (ERF) proteins such as AaERF1, AaERF2, AaORA and AaTAR1 have been identified as positive regulators, enhancing artemisinin synthesis by upregulating genes like *AaADS*, *AaCYP71AV1* and *AaDBR2* (Lu et al. 2013; Tan et al. 2015; Yu et al. 2012). Plant hormones, particularly jasmonate (JA), have been found to play significant regulatory roles. The glandular trichome‐specific WRKY transcription factor AaGSW1 binds to W‐box elements in the promoters of *AaCYP71AV1* and *AaORA*, facilitating their expression in response to JA (Chen et al. 2016). Similarly, AaMYC2, a basic helix‐loop‐helix TF responsive to JA, enhances artemisinin synthesis by binding directly to the G‐box in the *AaDBR2* promoter and activating the expressions of *AaCYP71AV1* and *AaDBR2* (Shen et al. 2016). Additionally, AaJAZ8, a JA pathway repressor, inhibits the transcriptional activity of the AaTCP14–AaORA complex, which activates the promoters of *AaDBR2* and *AaALDH1*. JA‐induced degradation of AaJAZ8 releases this inhibition, leading to increased artemisinin production (Ma et al. 2018). Collectively, these findings highlight the central role of JA signalling in modulating artemisinin biosynthesis in *A. annua* . Sunlight is crucial not only for photosynthesis and the overall longevity of plants but also plays an integral role in regulating various developmental and growth processes (Xing and Quail 1999). To adapt to light stimuli, plants have developed complex transcriptional networks mediated by photoreceptors, such as phytochromes, cryptochromes, phototropins and UVR8 (Jiao et al. 2007). For example, overexpression of *AtCRY1*, a blue light receptor gene from *Arabidopsis*, was shown to boost artemisinin production in *A. annua* by enhancing the transcription of *FPS*, *ADS* and *CYP71AV1* (Hong et al. 2009). Similarly, research conducted by Zhang et al. (2018) demonstrated that red and blue light can significantly increase artemisinin accumulation by activating the expression of its biosynthetic genes, with the highest levels recorded under red and blue light compared to white light, infrared and darkness. Our studies also demonstrated a marked reduction in the expression of artemisinin biosynthesis genes in darkness versus light conditions. Transcriptome analyses of *A. annua* leaves treated with methyl jasmonate (MeJA) under light and dark conditions revealed that JA signalling linked with light significantly enhances artemisinin biosynthesis (Hao et al. 2017). Moreover, the bZIP transcription factor ELONGATED HYPOCOTYL 5 (HY5) was identified as a critical component in this process, directly activating artemisinin biosynthesis by binding to the promoter of *AaGSW1* (Hao et al. 2019). Another important regulator, the R2R3‐MYB transcription factor AaMYB15, which is upregulated under both darkness and JA treatment, was found to negatively impact artemisinin biosynthesis by binding directly to the promoter of *AaORA* (Wu et al. 2021). The transcriptional regulation of artemisinin is intricately complex, with environmental factors and hormones interacting to modulate artemisinin synthesis such as light and JA (Fu et al. 2021; Hao et al. 2017; Liu et al. 2023; Ma et al. 2021). Transcriptome analysis unveiled AaWRKY9, specifically expressed in glandular trichomes, as a key facilitator of artemisinin accumulation by binding to the W‐box element within the *AaDBR2* and *AaGSW1* promoters. Yeast one‐hybrid and electrophoretic mobility shift assay experiments demonstrated that AaHY5 induces *AaWRKY9* expression. Additionally, AaWRKY9 interacts with AaJAZ9, with AaJAZ9 acting to suppress transcriptional activation of AaWRKY9. These findings suggest that AaWRKY9 orchestrates the upregulation of artemisinin biosynthesis induced by light and JA in *A. annua* (Fu et al. 2021). AaTCP15, a transcription factor responsive to both JA and abscisic acid (ABA), interacts with the positive regulator AaORA and directly enhances the expression of *AaDBR2* and *AaALDH1* by binding to their respective promoters. Overexpression of *AaTCP15* resulted in increased artemisinin production. Y1H and EMSA analyses further revealed that the dual JA‐ and ABA‐responsive protein AaGSW1 binds to the promoter and activates the expression of *AaTCP15* (Ma et al. 2021). Our group also identifies AaMYB108, a MYB transcription factor, as a crucial positive regulator of artemisinin biosynthesis in *A. annua* following light and JA treatment. AaMYB108 enhances artemisinin production by interacting with AaGSW1 and also interacted with AaCOP1 and AaJAZ8. Collectively, our results elucidate the molecular mechanism by which JA regulates artemisinin biosynthesis in response to light in *A. annua* (Liu et al. 2023).

And interestingly enough, previous studies have shown that both artemisinin biosynthetic enzyme genes and transcription factor genes regulating glandular trichome development and artemisinin biosynthesis are highly expressed in young leaves. As the leaves mature and eventually senesce, the expression levels of these genes decrease significantly. Notably, the expressions of both biosynthetic enzyme genes and those transcription factor genes decline sharply in the second and third leaves. For instance, qRT‐PCR analysis revealed that both wild‐type (*pALDH1*‐*ALDH1*) and recombinant *ALDH1* (*pALDH1*‐*GUS*) promoters showed peak activity in the youngest leaf, gradually declining with leaf maturation, aligning with the expression patterns of artemisinin biosynthetic genes (Liu et al. 2016; Wang et al. 2013). The expression patterns of *AaORA*, *AaGSW1*, *AaWRKY9* and *AaMYB108*, encoding the crucial positive regulators, are similar to those of artemisinin biosynthetic genes, showing high expression in very young leaves with rapid decline during development (Chen et al. 2016; Fu et al. 2021; Liu et al. 2023; Lu et al. 2013). In addition to artemisinin biosynthetic genes and transcription factor genes involved in positively regulating artemisinin biosynthesis, *AaGSW2*, *AaHD1*, *AaMIXTA1*, *AaHD8* and *AaSPL9*, encoding the transcriptional regulators governing the development of glandular trichomes, also exhibit analogous expression patterns (He et al. 2022; Shi et al. 2018; Xie et al. 2021b; Yan et al. 2017, 2018). These results indicate that a developmental regulation of artemisinin biosynthesis, tightly linked to leaf age, suggests that artemisinin is likely synthesised in young leaves and accumulated in mature leaves. The specific expression pattern raises the question of whether it is regulated by plant hormones. Despite these observations, the regulatory mechanisms underlying the positional and developmental control of artemisinin biosynthesis across the leaf gradient remain poorly understood. In this study, our study reveals a spatiotemporal regulatory network in *A. annua* , where AabHLH93 and AaWRKY9 function as synergistic transcriptional activators to drive artemisinin biosynthesis in young leaves, while AaMYB7 and AaJAZ9 act as antagonistic repressors in mature leaves, thereby orchestrating leaf‐age‐dependent artemisinin accumulation through dynamic activator‐repressor interplay.

## 2 Results

### 2.1 Identification of AabHLH93 as an AaWRKY9‐Interacting Protein

**FIGURE 1:**
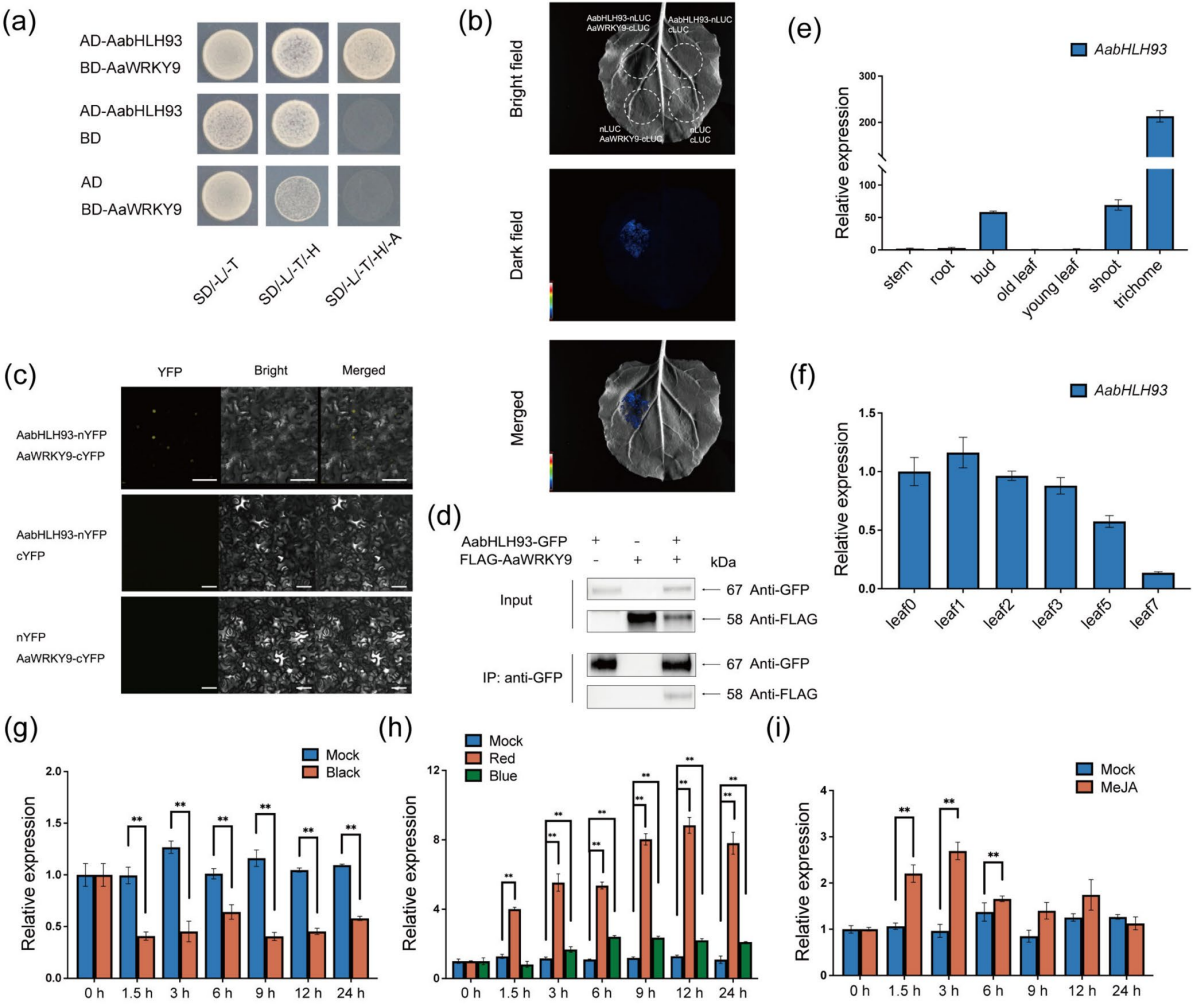
AabHLH93 Interacts with AaWRKY9 in vitro and in vivo. (a) Yeast two‐hybrid (Y2H) assays were conducted to investigate the interaction between AabHLH93 and AaWRKY9. Transformed yeast cells were cultured on control (SD/‐Trp/‐Leu) and selective media (SD/‐Trp/‐Leu/‐His and SD/‐Trp/‐Leu/‐His/‐Ade). Following a 4‐day incubation at 30°C, images were captured, with representative results from three independent experiments shown. (b) Luciferase complementation assays validated the AaWRKY9–AabHLH93 interaction through *Agrobacterium*‐mediated transient co‐expression in *Nicotiana benthamiana*. AabHLH93 and AaWRKY9 were translationally fused to the N‐terminal (nLUC) and C‐terminal (cLUC) luciferase fragments, respectively. Bioluminescence imaging revealed restored luciferase functionality exclusively in co‐expressed leaves, while negative controls expressing empty vectors exhibited background luminescence levels (three biological replicates; detection via low‐light CCD camera system). (c) Bimolecular fluorescence complementation (BiFC) was employed to validate the AaWRKY9–AabHLH93 interaction in *N. benthamiana* epidermal cells. Recombinant constructs were generated by fusing the N‐terminal YFP fragment (nYFP) to AabHLH93 and the C‐terminal YFP fragment (cYFP) to AaWRKY9. Fluorescence signal recovery confirmed complex formation in three biological replicates. Interaction between AaWRKY9 and AabHLH93 reconstituted luciferase activity, resulting in luminescence signals detected by a bioluminescence imaging system. Negative control plants, which received empty vector constructs, showed no detectable luminescence, confirming the specificity of the interaction. Three independent transfections were performed. (d) Co‐immunoprecipitation (Co‐IP) assays in *N. benthamiana* leaves demonstrated physical association between FLAG‐tagged AaWRKY9 and GFP‐tagged AabHLH93. Total protein lysates were subjected to immunoprecipitation using anti‐GFP magnetic beads, followed by immunoblotting with anti‐FLAG antibodies. Three independent experiments yielded consistent interaction patterns. (e) Tissue‐specific profiling of *AabHLH93* transcript accumulation in *Artemisia annua* revealed the expression patterns in the different tissues. Expression levels of *AabHLH93* across stem, root, old leaf, young leaf, shoot, bud, and trichome tissues are normalised to stem expression values. (f) Developmental regulation of *AabHLH93* transcript accumulation was analysed across leaf ontogeny in *A. annua*. Leaf0 (meristem), leaf1 (first leaf below meristem), leaf2, leaf3, leaf5, and leaf7 were collected from the main stem of 5‐month‐old *A. annua* plants, with Leaf0 serving as the reference baseline. (g, h) Photobiological regulation of *AabHLH93* expression in *A. annua* plants. Expression levels of *AabHLH93* (g) in darkness and exposed to (h) monochromatic blue light (40 μmol m^−2^ s^−1^) and monochromatic red light (40 μmol m^−2^ s^−1^) following 24‐h dark acclimation were detected. White light was used as a mock treatment. (i) Expression levels of *AabHLH93* were further assessed under 100 μM methyl jasmonate (MeJA) treatment, with equivalent ethanol concentrations (0.1%) as the vehicle control. All data were standardised against *Actin* reference transcripts and derived from triplicate biological experiments (mean ± SD). Asterisks indicate statistically significant divergence between the treated groups and control groups (**p < 0.01; Student's *t*‐test).

To further explore the exact mechanism of regulating artemisinin biosynthesis, AaWRKY9 was employed as bait in a yeast two‐hybrid (Y2H) screen to identify its interacting proteins. The screen utilised an *A. annua* cDNA library constructed from young leaves and meristems. A bHLH protein, AabHLH93, was identified as an interacting partner of AaWRKY9. The interaction between AaWRKY9 and AabHLH93 protein was confirmed using Y2H (Figure 1a). The physical interaction between AaWRKY9 and AabHLH93 was additionally validated through the luciferase complementation assays, BiFC and Co‐IP assays in *Nicotiana benthamiana* leaf cells in vivo. In luciferase complementation assays, AabHLH93 and AaWRKY9 were fused to the N‐terminal (nLUC) and C‐terminal (cLUC) fragments of the firefly luciferase (LUC) gene, respectively. After the constructs were co‐transformed into *N. benthamiana* leaves, the interaction between AaWRKY9 and AabHLH93 reconstituted the luciferase enzyme, producing a detectable luminescence signal (Figure 1b). BiFC assays were also performed to confirm this interaction between AaWRKY9 and AabHLH93. When these constructs (*AabHLH93*‐*nYFP* and *AaWRKY9‐cYFP*) were co‐expressed in *N. benthamiana* cells, the fluorescence signal was detected using confocal microscopy, indicating that AaWRKY9 interacted with AabHLH93 in plants (Figure 1c). Furthermore Co‐IP assays also indicated that Flag‐AaWRKY9 proteins were immunoprecipitated with AabHLH93‐GFP proteins (Figure 1d). These results provided strong biochemical evidence for the physical interaction between AaWRKY9 and AabHLH93.

### 2.2 *AabHLH93* Exhibits Tissue‐Specific Expression Patterns Consistent With Artemisinin Biosynthesis

To explore the function of *AabHLH93* in *A. annua* , the expression patterns of *AabHLH93* were investigated in different tissues of *A. annua* . qRT‐PCR analysis revealed that *AabHLH93* was predominantly expressed in trichomes, and its expression was also detected in buds and shoots (Figure 1e). This is consistent with the fact that glandular trichomes are the primary sites of artemisinin biosynthesis and accumulation. Furthermore, *AabHLH93* expression exhibited a dynamic pattern during leaf development. Transcript levels were highest in young leaves and gradually decreased as leaves matured (Figure 1f). The tissue‐specific and developmentally regulated expression pattern of *AabHLH93*, which closely mirrors the expression patterns of artemisinin biosynthetic genes and regulatory transcription factors, strongly supports its role as a key regulator of artemisinin biosynthesis in *A. annua* . Interestingly, *AabHLH93* expression exhibited a distinct diurnal rhythm under light/dark cycles, with lower transcript levels detected during the dark period compared to the light period (Figure 1g). AabHLH93 transcript levels exhibited a strikingly similar expression pattern to these artemisinin biosynthetic genes, including *AaADS*, *AaCYP71AV1*, *AaDBR2* and *AaALDH1*. To investigate the expression patterns of *AabHLH93* under varying light conditions, we examined its expression after a dark adaptation followed by exposure to blue or red light. qRT‐PCR analysis revealed that *AabHLH93* expression was rapidly and significantly induced upon exposure to both blue and red light (Figure 1h). Besides, *AabHLH93* transcript levels were significantly upregulated in response to MeJA treatment, with a peak expression observed at 3 h post‐treatment (Figure 1i). Besides, a phylogenetic tree analysis revealed that AabHLH93 clustered within the same clade as HabHLH93 from *Helianthus annuus* , NtbHLH93 from *N. tabacum* , AabHLH113 from *A. annua* (Yuan et al. 2023) and AtbHLH93 from *Arabidopsis thaliana* (Figure S1a). Multiple sequence alignment of AabHLH93 with its homologues from other plant species revealed a high degree of conservation within the bHLH domain, indicating that the core residues of the bHLH domain, including the basic region responsible for DNA binding and the helix‐loop‐helix region involved in protein–protein interactions, were highly conserved across species (Figure S1b). To investigate the regulatory role of AabHLH93, a 1654 bp fragment upstream of the *AabHLH93* coding sequence was cloned and fused to the β‐glucuronidase (GUS) reporter gene. This construct was then transformed into *A. annua* plants. Histochemical GUS staining revealed that the *AabHLH93* promoter drives strong GUS expression in glandular secretory trichomes (GSTs) and non‐glandular trichomes (TSTs) (Figure S1c), consistent with the qRT‐PCR results showing high *AabHLH93* expression in trichomes. To determine its cellular distribution, we transiently expressed a 35S::AabHLH93‐YFP fusion construct in *N. benthamiana* epidermal cells. Subcellular localization analysis revealed that AabHLH93 is predominantly located in the nucleus (Figure S2a). This trichome‐specific expression pattern further supports the hypothesis that AabHLH93 plays a crucial role in regulating trichome‐specific processes, including artemisinin biosynthesis. These results clearly demonstrate that AabHLH93 is a nuclear‐localized protein, consistent with its predicted function as a transcription factor in regulating artemisinin biosynthesis. In summary, these results strongly support the role of AabHLH93 in integrating MeJA and light signals to regulate artemisinin biosynthesis in *A. annua* .

### 2.3 AabHLH93 Positively Regulates Artemisinin Content Through Direct Binding to the *CYP71AV1* Promoter in *Artemisia annua*

**FIGURE 2:**
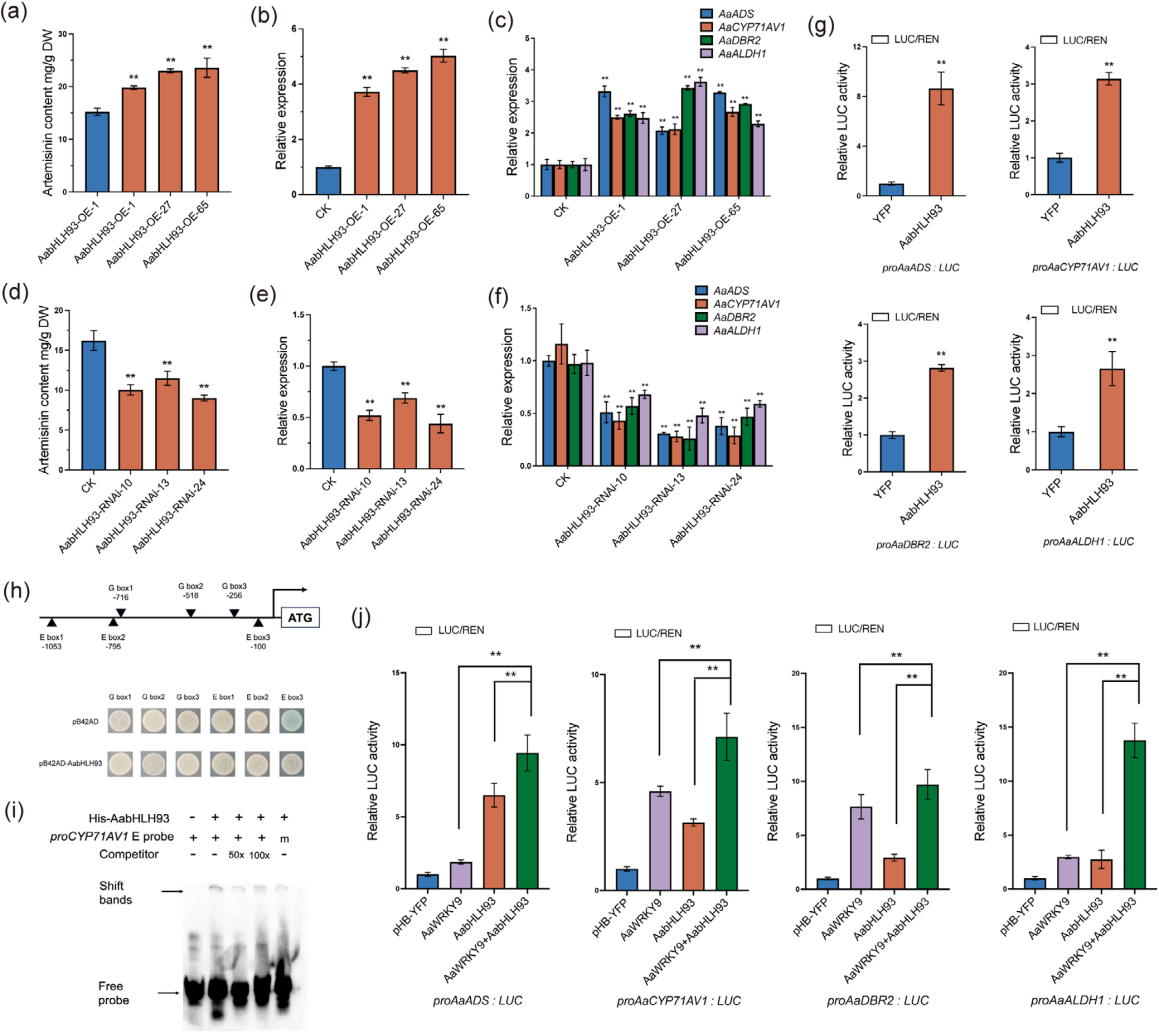
AabHLH93 Positively regulates artemisinin biosynthesis through directly binding to the E3‐box of *AaCYP71AV1* promoter in *A. annua*. (a, d) The artemisinin contents were measured in overexpression (a), *AabHLH93*‐RNAi (d) transgenic *A. annua* plants, and wild type *A. annua* plants (CK). All contents are comparisons relative to the contents of wild type *A. annua* plants. (b, e) Expression levels of *AabHLH93* were measured in overexpression (b), *AabHLH93*‐RNAi (e) transgenic *A. annua* plants, and wild type *A. annua* plants (CK). Transcript levels were normalised to wild type *A. annua* plants. (c, f) *Actin*‐normalised expression of artemisinin pathway genes (*AaADS*, *AaCYP71AV1*, *AaDBR2*, and *AaALDH1*) were measured in overexpression (c), *AabHLH93*‐RNAi (f) transgenic *A. annua* plants, and wild type *A. annua* plants (CK). *Actin* was used as an internal control. (g) Dual‐Luciferase reporter assays demonstrate that AabHLH93 activates the expressions of *AaADS*, *AaCYP71AV1*, *AaDBR2* and *AaALDH1*. Firefly luciferase activity was normalised to Renilla luciferase activity (Fluc/Rluc ratio). (h) Y1H assays showing that AabHLH93 binds to the E box3 of *AaCYP71AV1*. Schematic representations illustrate the promoter architectures of *AaCYP71AV1* promoter. Putative G box and E box motifs are annotated as triangles, with positional numbering reflecting their genomic coordinates relative to the designated translational initiation site (ATG, +1). Yeast EGY48A strains transformed with recombinant pB42AD vectors (empty or pB42AD‐*AabHLH93*) and pLacZ vectors (pLacZ‐G box motifs or pLacZ‐E box motifs from *AaCYP71AV1* promoter) were cultured at 30°C for 96 h on selection medium (SD/‐Trp/‐Ura) supplemented with X‐gal (20 mg/L) as a β‐galactosidase substrate. Protein‐DNA binding activity was detected via chromogenic conversion (blue colony formation). Experiments included three biological replicates, with representative outcomes displayed. (i) EMSA assays showing that AabHLH93 binds to the E box3 of *AaCYP71AV1*. Competitive binding reactions incorporated unlabeled probe as a specificity competitor at increasing molar excess gradients (50×, 100×) relative to the labelled counterpart. His‐TF protein was used as a negative control. ‘m’ means mutation probe. (j) Dual‐Luciferase reporter assays demonstrate that AaWRKY9 and AabHLH93 synergistically activates the expressions of *AaADS*, *AaCYP71AV1*, *AaDBR2* and *AaALDH1*. The YFP effector in the mock treatment served as a negative control. Three independent transfection experiments were performed. Data represent the means ± SD from three biological replicates. Student's *t*‐test: **p < 0.01.

**FIGURE 3:**
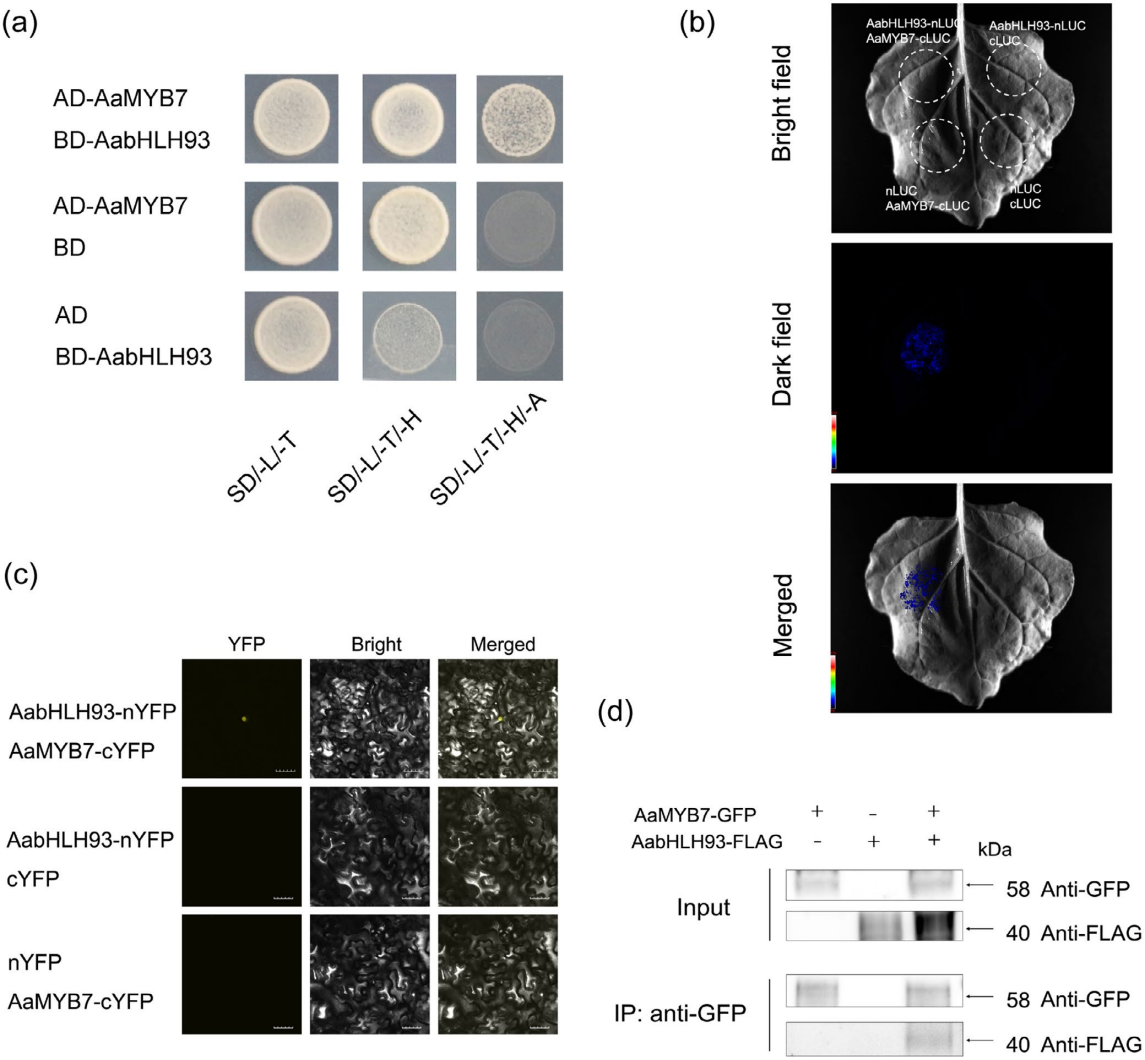
AabHLH93 interacts with AaMYB7 in vitro and in vivo. (a) Protein interaction analysis between AabHLH93 and AaMYB7 was conducted using a Y2H system. Transformed yeast colonies were grown on the selective media (SD/‐Trp/‐Leu/‐His and SD/‐Trp/‐Leu/‐His/‐Ade). Cultures were incubated at 30°C for 96 h before documentation. Y2H assays were repeated three times, and representative results are shown. (b) Luciferase complementation assays validated the interaction of AabHLH93 and AaMYB7 through *Agrobacterium*‐mediated transient co‐expression in *N. benthamiana*. AabHLH93 and AaMYB7 were respectively fused to the N‐terminal (nLUC) and C‐terminal (cLUC) luciferase fragments. Bioluminescence imaging revealed restored luciferase functionality exclusively in co‐expressed leaves, while negative controls expressing empty vectors exhibited background luminescence levels (three biological replicates; detection via low‐light CCD camera system). (c) BiFC was employed to validate the interaction of AabHLH93 and AaMYB7 in *N. benthamiana* epidermal cells. Recombinant constructs were generated by fusing the N‐terminal YFP fragment (nYFP) to AabHLH93 and the C‐terminal YFP fragment (cYFP) to AaMYB7. Interaction between AabHLH93 and AaMYB7 reconstituted luciferase activity, resulting in luminescence signals detected by a bioluminescence imaging system. Negative control plants, which received empty vector constructs, showed no detectable luminescence, confirming the specificity of the interaction. Three independent transfections were performed. (d) Co‐IP assays in *N. benthamiana* leaves demonstrated physical association between FLAG‐tagged AabHLH93 and GFP‐tagged AaMYB7. Total protein lysates were subjected to immunoprecipitation using anti‐GFP magnetic beads, followed by immunoblotting with anti‐FLAG antibodies. Three independent experiments yielded consistent interaction patterns.

To elucidate the functional role of AabHLH93 in artemisinin biosynthesis, we generated transgenic *A. annua* lines with either overexpression or RNAi‐mediated suppression of *AabHLH93*. The overexpression of *AabHLH93* led to a significant enhancement in artemisinin content compared to wild‐type controls, demonstrating the positive regulatory role of AabHLH93 in artemisinin biosynthesis (Figure 2a,b). Conversely, RNAi‐mediated suppression of *AabHLH93* resulted in a marked reduction in artemisinin accumulation, further confirming the critical involvement of *AabHLH93* in the regulation of artemisinin production (Figure 2d,e). In *AabHLH93*‐overexpressing plants, the transcript levels of key genes, including *AaADS*, *AaCYP71AV1*, *AaDBR2* and *AaALDH1*, were significantly upregulated compared to wild‐type controls (Figure 2c). In contrast, in *AabHLH93*‐RNAi suppression lines, the expression of these genes was markedly downregulated (Figure 2f). These findings collectively suggest that AabHLH93 acts as a positive transcriptional regulator promoting artemisinin biosynthesis in *A. annua* . In addition to the transgenic analysis, we conducted further molecular assays to elucidate the regulatory mechanism of AabHLH93. Dual‐luciferase (dual‐LUC) reporter assays demonstrated that AabHLH93 significantly activated the promoters of the artemisinin biosynthetic genes, including *AaADS*, *AaCYP71AV1*, *AaDBR2* and *AaALDH1*, confirming its role as a transcriptional activator (Figure 3g). Yeast one‐hybrid assays confirmed the direct interaction between AabHLH93 and the E box3 in the *AaCYP71AV1* promoter, as evidenced by the growth of transformants on the selective media with β‐galactosidase (Figure 3h). Furthermore, electrophoretic mobility shift assays (EMSA) revealed the specific binding of recombinant AabHLH93 protein to the E‐box *cis*‐element in the *AaCYP71AV1* promoter (Figure 3i). Collectively, these results demonstrate that AabHLH93 directly activates *AaCYP71AV1* expression by directly binding to its promoter, thereby positively regulating artemisinin biosynthesis.

### 2.4 Synergistic Interaction of AabHLH93 and AaWRKY9 Enhances Transcriptional Regulation of Artemisinin Biosynthesis

To further investigate the synergistic regulatory effects of AabHLH93 and AaWRKY9 on artemisinin biosynthesis, a dual‐LUC reporter assay was performed. When *AabHLH93* or *AaWRKY9* was expressed alone, a moderate increase in LUC/REN activity was observed, indicating that both transcription factors could independently activate the promoters of artemisinin biosynthetic enzyme genes (Figure 3j). However, when *AabHLH93* and *AaWRKY9* were co‐expressed, the LUC/REN activity was significantly enhanced compared to the individual effects, indicating a synergistic interaction between the two transcription factors in regulating artemisinin biosynthesis (Figure 3j). Besides, the dual‐LUC reporter assay also indicated that AaWRKY9 could activate the promoter of *AabHLH93* in transiently transfected *N. benthamiana* leaves, and co‐expression of *AaJAZ9* and *AaWRKY9* demonstrated that AaJAZ9 suppresses AaWRKY9‐mediated transcriptional activation of the *AabHLH93* promoter (Figure S3). These results suggest a potential regulatory loop in which AaWRKY9 not only activates the promoter of *AabHLH93* but also physically interacts with AabHLH93 to synergistically amplify the transcriptional activation of artemisinin biosynthetic enzyme genes to robustly boost artemisinin biosynthesis under JA and light signalling.

### 2.5 Identification of AaMYB7 as an AabHLH93‐Interacting Protein

To further explore the regulatory mechanisms underlying artemisinin biosynthesis, we used AabHLH93 as bait in a Y2H screening to identify potential interacting proteins. Through this screening, we identified AaMYB7 as a direct interacting partner of AabHLH93. The physical interaction between AaMYB7 and AabHLH93 was confirmed through Y2H assays, where co‐expression of AaMYB7 and AabHLH93 in yeast cells resulted in robust growth on selective media, indicating a direct interaction (Figure 3a). To validate this interaction in planta, we performed luciferase complementation assays by fusing AabHLH93 to nLUC and AaMYB7 to cLUC. Co‐expression of these constructs in *N. benthamiana* leaves reconstituted luciferase activity, producing a detectable luminescence signal (Figure 3b). Additionally, BiFC assays also confirmed the interaction between AaMYB7 and AabHLH93 in vivo (Figure 3c). Furthermore Co‐IP assays demonstrated that AabHLH93‐Flag proteins could be immunoprecipitated with AaMYB7‐GFP proteins, providing additional biochemical evidence for their physical interaction (Figure 3d). These results collectively demonstrate that AaMYB7 and AabHLH93 form a protein complex.

### 2.6 JA Repressed AaMYB7 Negatively Regulates Artemisinin Biosynthesis in *Artemisia annua*

**FIGURE 4:**
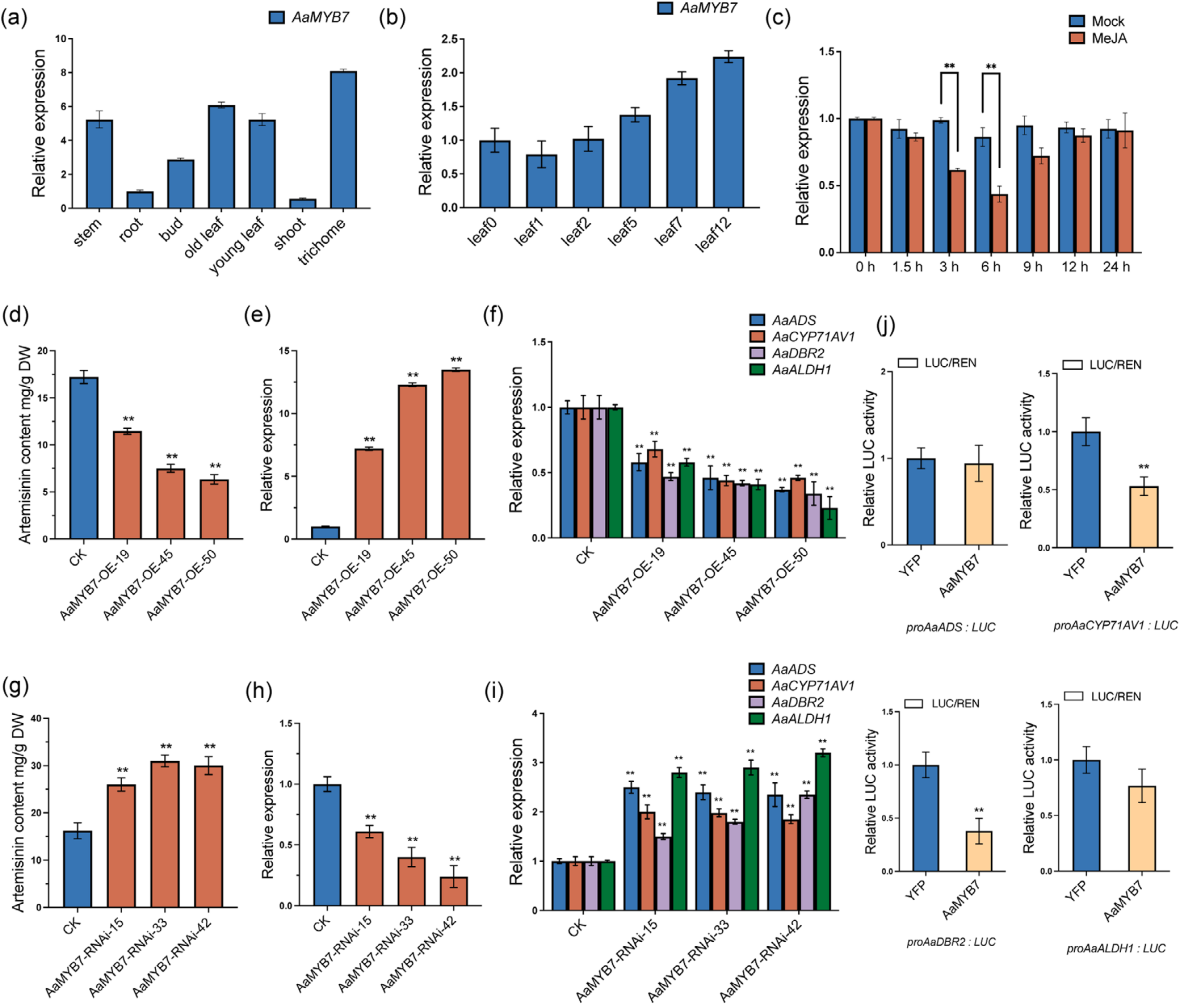
AaMYB7 negatively regulates artemisinin biosynthesis. (a) Tissue‐specific profiling of *AaMYB7* transcript accumulation in *A. annua* revealed the expression patterns in the different tissues. Expression levels of *AaMYB7* across stem, root, old leaf, young leaf, shoot, bud and trichome tissues are normalised to stem expression values. (b) Developmental regulation of *AaMYB7* transcript accumulation was analysed across leaf ontogeny in *A. annua*. Leaf0 (meristem), leaf1 (first leaf below meristem), leaf2, leaf5, leaf7 and leaf12 were collected from the main stem of 5‐month‐old *A. annua* plants, with Leaf0 serving as the reference baseline. (c) Expression levels of *AaMYB7* were further assessed under 100 μM MeJA treatment, with equivalent ethanol concentrations (0.1%) as the control. All data were standardised against *Actin* reference transcripts. (d, g) Expression levels of *AaMYB7* were measured in overexpression (d), *AaMYB7‐*RNAi (g) transgenic *A. annua* plants, and wild type *A. annua* plants (CK). Transcript levels were normalised to wild type *A. annua* plants. (e, h) The artemisinin contents were measured in overexpression (e), *AaMYB7‐*RNAi (d) transgenic *A. annua* plants, and wild type *A. annua* plants (CK). All contents are comparisons relative to the contents of wild type *A. annua* plants. (f, i) *Actin*‐normalised expression of artemisinin pathway genes (*AaADS*, *AaCYP71AV1*, *AaDBR2* and *AaALDH1*) were measured in overexpression (f), *AaMYB7‐*RNAi (i) transgenic *A. annua* plants, and wild type *A. annua* plants (CK). *Actin* was used as an internal control. Asterisks denote a significant difference of *AaMYB7* transgenic lines relative to wild type plant as determined by a Student's *t*‐test: **p < 0.01. (j) Dual‐Luciferase reporter assays demonstrate that AaMYB7 inhibits the expressions of *AaCYP71AV1* and *AaDBR2*. Firefly luciferase activity was normalised to Renilla luciferase activity (Fluc/Rluc ratio). Three independent transfection experiments were performed. Data represent the means ± SD from three biological replicates. Student's *t*‐test: **p < 0.01.

Similar to the artemisinin biosynthetic enzyme genes, *AaMYB7* exhibited high expression levels in trichomes (Figure 4a), the primary sites of artemisinin production. However, unlike the biosynthetic genes, which typically show decreased expression during leaf development, *AaMYB7* expression increased as leaves matured, suggesting a potential regulatory role on the artemisinin biosynthesis in the matured leaves of *A. annua* (Figure 4b). Furthermore, *AaMYB7* expression was significantly suppressed by MeJA treatment (Figure 4c). Subcellular localization studies revealed that AaMYB7 is predominantly located in the nucleus of *N. benthamiana* cells, consistent with its role as a transcription factor (Figure S2b). To elucidate the evolutionary relationship and functional motifs of AaMYB7, a phylogenetic tree was constructed with AaMYB7 and representative plant R2R3‐MYB proteins (Figure S4a). The results revealed that AaMYB7 clustered with the reported repressors AtMYB4 and AtMYB7 from *Arabidopsis*, suggesting its potential role as a repressor in secondary metabolism (Figure S4a). Multiple sequence alignment further demonstrated that AaMYB7 contained a conserved N‐terminal R2R3‐MYB domain and EAR (ERF‐associated amphiphilic repression) motif (LxLxL) (Ohta et al. 2001), further suggesting that AaMYB7 functioned as negative regulators of gene expression (Figure S4b). Notably, histochemical GUS staining driven by the *AaMYB7* promoter showed specific expression in both the glandular trichomes and T‐shaped trichomes of *A. annua* (Figure S4c), consistent with its putative function in artemisinin biosynthesis. These findings suggest that AaMYB7 may act as a negative regulator of artemisinin biosynthesis in mature leaves. To confirm the negative regulatory role of AaMYB7 in artemisinin biosynthesis, transgenic *A. annua* plants overexpressing or silencing *AaMYB7* can be generated. HPLC quantification revealed that artemisinin content in leaves of *AaMYB7* overexpression lines decreased by 33%–63% compared to WT, while RNAi lines showed a 1.6‐ to 1.8‐fold increase in artemisinin accumulation (Figure 4d,g). qRT‐PCR analysis confirmed that *AaMYB7* expression in overexpressing lines was 7.2‐ to 13.5‐fold higher than in wild‐type (WT) plants, whereas RNAi lines exhibited 39%–76% reduction in *AaMYB7* transcripts (Figure 4e,h). Transcriptome profiling further demonstrated that key artemisinin biosynthetic genes were significantly downregulated in *AaMYB7* overexpression lines but upregulated in RNAi lines (Figure 4f,i). These genetic and molecular data collectively demonstrate that AaMYB7 acts as a transcriptional repressor to suppress artemisinin biosynthesis by inhibiting the expression of pathway‐specific genes.

### 2.7 AaJAZ9 and AaMYB7 Exert Dominant‐Negative Suppression on the Transcriptional Activation Complex Formed by AabHLH93 and AaWRKY9

**FIGURE 5:**
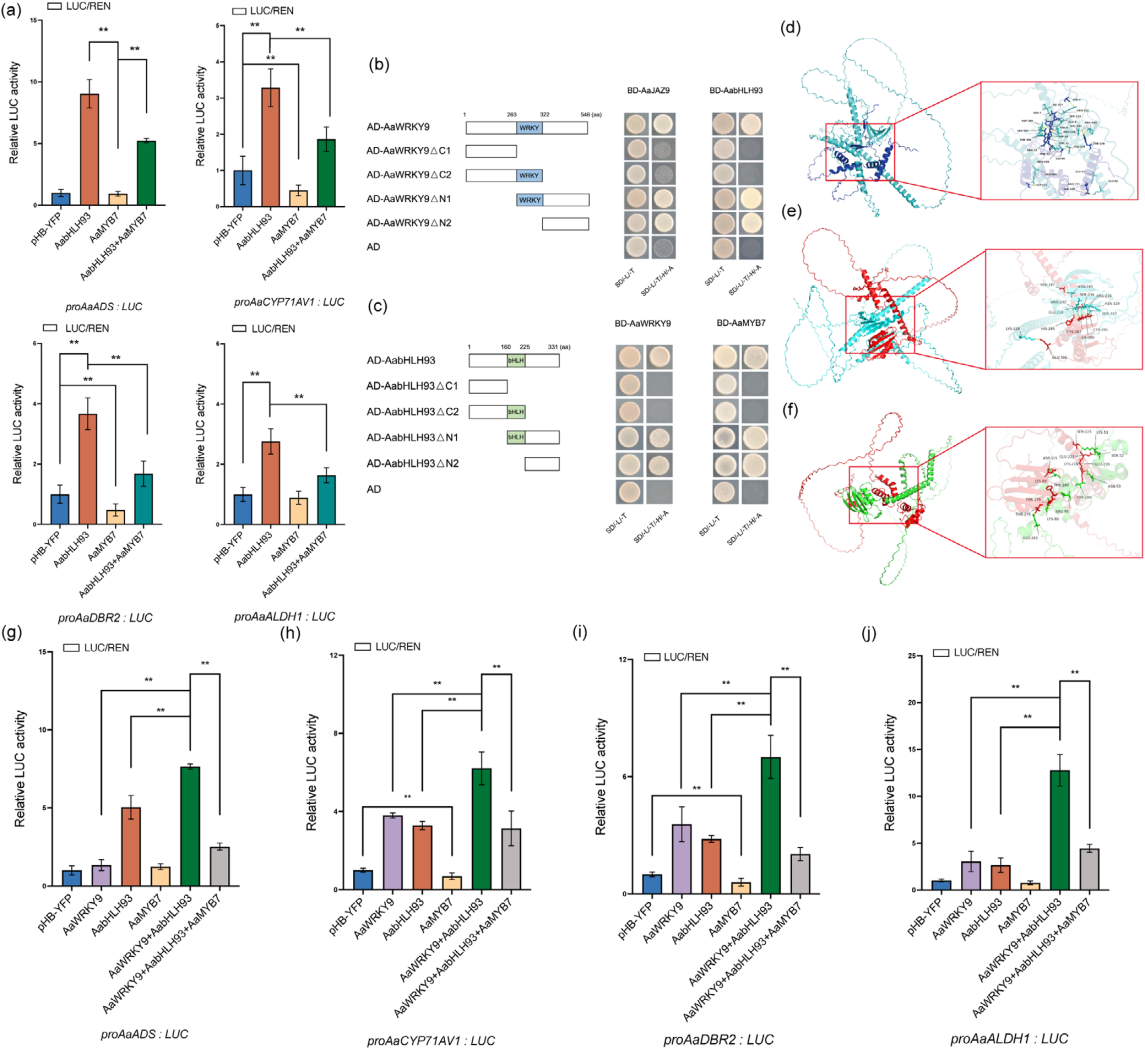
AaMYB7 interferes with the interaction between AaWRKY9 and AabHLH93, and attenuates the transcriptional activation activity of AaWRKY9 and AabHLH93. (a) Dual‐Luciferase reporter assays demonstrate that AaMYB7 represses the transcriptional activation function of AabHLH93. The YFP effector in the mock treatment served as a negative control. (b) Y2H analysis showing the interactions between AaJAZ9, AabHLH93, and full‐length and truncated versions of AaWRKY9. The schematic representations of AaWRKY9 truncation variants are on the left. Blue box represents the conserved domain of AaWRKY9. Right, transformed yeast colonies were grown on the selective media. Cultures were incubated at 30°C for 96 h before documentation. (c) Y2H analysis showing the interactions between AaWRKY9, AaMYB7, and full‐length and truncated versions of AabHLH93. The schematic representations of AabHLH93 truncation variants are on the left. Green box represents the conserved domain of AabHLH93. Right, transformed yeast colonies were grown on the selective media. Cultures were incubated at 30°C for 96 h before documentation. (d–f) AlphaFold3‐predicted interfacial residues (sphere representation) for three interacting pairs: AaJAZ9–AaWRKY9 (blue/cyan), AaWRKY9–AabHLH93 (cyan/red), AabHLH93–AaMYB7 (red/green). (g–j) Dual‐Luciferase reporter assays demonstrate that AaMYB7 represses the transcriptional activation activity of AaWRKY9 and AabHLH93. The YFP effector in the mock treatment served as a negative control. Three independent transfection experiments were performed. Data represent the means ± SD from three biological replicates. Student's *t*‐test: **p < 0.01.

To investigate the regulatory role of AaMYB7 in artemisinin biosynthesis, dual‐LUC reporter assays were performed in *N. benthamiana* leaves. The promoters of *AaADS*, *AaCYP71AV1*, *AaDBR2* and *AaALDH1* were fused to LUC as the reporter with REN for normalisation; *AaMYB7* was cloned into the effector plasmid under the CaMV 35S promoter. The results showed that co‐expression of *AaMYB7* with the promoters of artemisinin biosynthetic genes (*AaCYP71AV1* and *AaDBR2*) resulted in a 47%–63% reduction in LUC activity compared to empty vector controls (Figure 4j), demonstrating its potent repressive activity. To further dissect the functional interplay between AabHLH93 (activator) and AaMYB7 (repressor) in regulating artemisinin biosynthesis, we performed dual‐LUC assays by co‐expressing both factors with the promoters of *AaADS*, *AaCYP71AV1*, *AaDBR2* and *AaALDH1*. Transient expression of *AabHLH93* alone in *N. benthamiana* leaves robustly activated the LUC activities of the promoters of *AaADS*, *AaCYP71AV1*, *AaDBR2* and *AaALDH1* (Figure 5a). Strikingly, co‐expression of *AaMYB7* with *AabHLH93* observably suppressed this activation, indicating that AaMYB7 functioned as a transcriptional repressor to antagonise AabHLH93‐mediated transcriptional activation of artemisinin biosynthetic genes through direct protein–protein interference, with the EAR motif serving as an essential determinant of AaMYB7's suppressive capacity (Figure 5a). The dynamic equilibrium between these factors likely underpins the developmental phase specificity of artemisinin accumulation in *A. annua* glandular trichomes. To unravel the molecular basis of this regulation, Y2H assays were conducted using truncated versions of AaWRKY9 and AabHLH93. Yeast two‐hybrid analysis revealed that the full‐length AaWRKY9 and the C‐terminal domain of AaWRKY9 independent of the WRKY DNA‐binding domain were sufficient to bind to AaJAZ9, while the N‐terminal WRKY domain showed no detectable interaction (Figure 5b). Intriguingly, both full‐length AaWRKY9 and its C‐terminal domain (AaWRKY9ΔN1 and AaWRKY9ΔN2) exhibited strong interaction signals with AabHLH93 in Y2H assays (Figure 5b). Notably, the C‐terminal domain of AabHLH93 mediated the physical interactions with AaWRKY9 and AaMYB7 independent of its conserved bHLH motif (Figure 5c). These results suggested that AaJAZ9 might sterically hinder AabHLH93 binding to AaWRKY9 through overlapping interaction surfaces, effectively repressing the AaWRKY9–AabHLH93 activation module. The simultaneous binding of AaWRKY9 and AaMYB7 to AabHLH93's C‐terminal domain implied that AaMYB7 could counterbalance the activation signal of AaWRKY9–AabHLH93 via its repressive activity. To further resolve the structural basis of the observed transcriptional interplay, we employed AlphaFold3 to predict the interaction interfaces between AaWRKY9–AaJAZ9, AaWRKY9–AabHLH93 and AabHLH93–AaMYB7. Structural modelling revealed that both AaJAZ9 and AabHLH93 specifically docked to the C‐terminal domain of AaWRKY9 (residues 232–460) (Figure 5d,e), while AaWRKY9 and AaMYB7 exhibited distinct binding affinities for the C‐terminal domain of AabHLH93 (residues 287–315) (Figure 5e,f). Notably, the predicted interaction surfaces between AaJAZ9/AabHLH93 and the C‐terminus of AaWRKY9 overlapped spatially, consistent with their mutually exclusive binding patterns observed in yeast two‐hybrid assays. To further elucidate the combinatorial regulation of artemisinin biosynthesis, we performed dual‐luciferase assays by transiently co‐expressing *AabHLH93*, *AaWRKY9* and *AaMYB7* individually or in combination in *N. benthamiana* leaves. Dual‐LUC assays demonstrated that *AaWRKY9* or *AabHLH93* alone activated the promoters of artemisinin biosynthetic genes, while *AaMYB7* alone showed no intrinsic activation capacity (Figure 5d–g). Co‐expression of *AaWRKY9* and *AabHLH93* synergistically activated the promoters of artemisinin biosynthetic genes, with combinatorial co‐expression inducing super additive transcriptional activation (Figure 5d–g). However, this synergistic activation was significantly attenuated by AaMYB7 (Figure 5d–g). Notably, the ternary combination incorporating *AaMYB7* with *AaWRKY9* and *AabHLH93* unexpectedly produced a contrasting effect—the LUC/REN ratio measured in *N. benthamiana* leaves showed 49%–71% significant reductions compared to the AaWRKY9–AabHLH93 complex (Figure 5d–g), suggesting that AaMYB7 functioned as a transcriptional suppressor modulating the synergistic activation complex.

## 3 Discussion

Artemisinin, a sesquiterpene lactone with unparalleled antimalarial efficacy, is synthesised in glandular trichomes of *A. annua* through a tightly regulated metabolic pathway (Posner et al. 2004; Thomas et al. 2008; van Agtmael et al. 1999; Wang et al. 2013). Its biosynthesis is dynamically modulated by both developmental programs and environmental stimuli (Chen et al. 2016; Fu et al. 2021; Hao et al. 2019; Liu et al. 2023; Ma et al. 2021; Shen et al. 2016; Shi et al. 2018; Xie et al. 2021a, 2021b; Yan et al. 2017; Zhang et al. 2015), yet the molecular mechanisms underlying this spatiotemporal control have remained incompletely resolved. Our study unveils a sophisticated transcriptional regulatory network governing artemisinin biosynthesis in *A. annua* , orchestrated by the dynamic interplay of activator–repressor modules. The identification of AaWRKY9–AabHLH93 as a central hub integrating light, JA signalling and transcriptional co‐regulation provides a paradigm for understanding how plants balance metabolic flux.

### 3.1 AabHLH93 as a Key Regulator in Artemisinin Biosynthesis Integrating Light and JA Signalling

In our previous studies, we identified AaWRKY9 as a key transcription factor involved in the regulation of artemisinin biosynthesis in *A. annua* . This glandular trichome‐specific WRKY protein was shown to directly activate the expression of critical biosynthetic genes *AaDBR2* and *AaGSW1* through binding to their promoter W‐box elements, establishing its fundamental role in modulating artemisinin production. We also demonstrate that AaWRKY9's transcriptional activity is dually regulated by the light‐responsive factor AaHY5 and the JA signalling component AaJAZ9 (Fu et al. 2021). In this study, we performed Y2H screening using AaWRKY9 as bait and identified AabHLH93 as its interacting partner. Y2H assays, luciferase complementation assays, BiFC and Co‐IP assays further confirmed this direct protein–protein association (Figure 1a–d). Our findings position AabHLH93 as a trichome‐enriched bHLH transcription factor with multifaceted regulatory potential in artemisinin biosynthesis (Figure S1c). The spatial–temporal expression pattern of *AabHLH93*, showing preferential accumulation in glandular trichomes and young leaves, coupled with developmental downregulation, aligns precisely with the biosynthesis dynamics of artemisinin and its key biosynthetic genes (Figure 1e,f). This spatial congruence strongly suggests its specialised role in glandular secretory cells where artemisinin is synthesised and stored. The observed light‐responsive regulation mirrors the known light dependency of artemisinin biosynthesis (Figure 1g,h). Notably, the JA‐responsive upregulation of *AabHLH93* parallels the established JA‐mediated enhancement of artemisinin production, suggesting its involvement in hormone signalling integration (Figure 1i). Collectively, these results also demonstrate striking similarities with other transcription factors regulating artemisinin biosynthesis, such as AaWRKY9 (Fu et al. 2021) and AaMYB108 (Liu et al. 2023), suggesting that AabHLH93 might be a strategic regulatory node potentially coordinating light signals and JA pathways to optimise artemisinin biosynthesis. The modulation of artemisinin content through overexpression and RNAi suppression provides unequivocal genetic evidence for its central regulatory role (Figure 2a,b,d,e). The upregulation/downregulation of core artemisinin biosynthetic genes in transgenic lines demonstrates that AabHLH93 potentially regulates multiple artemisinin biosynthetic genes (Figure 2e,f). The dual‐LUC assays also show AabHLH93 activates the expression of artemisinin biosynthetic enzyme genes (Figure 2g). Y1H and EMSA assays conclusively demonstrate that AabHLH93 directly targets the E‐box3 element in the *AaCYP71AV1* promoter (Figure 2h,i). Our findings demonstrate that AabHLH93 serves as a pivotal transcriptional activator governing artemisinin biosynthesis in *A. annua* .

### 3.2 AaMYB7 Exhibits Trichome‐Specific, Developmentally Regulated Expression and Suppresses Artemisinin Accumulation

As an R2R3‐MYB protein containing a repression domain EAR motif (Figure S4b), AaMYB7 was identified through Y2H screening using AabHLH93 as bait. Protein–protein interaction was further validated through Y2H, luciferase complementation, BiFC and Co‐IP assays (Figure 3a–d). The EAR motif recruits corepressors, such as TOPLESS (TPL), to suppress the transcription of target genes (Kagale and Rozwadowski 2011). Dual‐luciferase reporter assays revealed that AaMYB7 represses the promoters of key biosynthetic genes (*AaCYP71AV1* and *AaDBR2*) (Figure 4j). Functional characterisation of transgenic *A. annua* lines also demonstrated that *AaMYB7* negatively regulates artemisinin biosynthesis (Figure 4d–i). Spatiotemporal expression profiling revealed that *AaMYB7* exhibited pronounced expression in glandular trichomes (Figure 4a), the primary site of artemisinin biosynthesis. Intriguingly, *AaMYB7* expression levels escalated during leaf maturation (Figure 4b), inversely correlating with the developmental decline of artemisinin biosynthetic genes. While our data robustly demonstrate that AaMYB7 exhibits its highest expression and exerts its most prominent regulatory influence in mature leaves, its presence in young leaves warrants consideration. Young leaves represent sites of active precursor DHAA biosynthesis. AaMYB7 might act as a ‘balancing factor’ during this early developmental stage to prevent the potential feedback inhibition because of the excessive accumulation of DHAA. Notably, *AaMYB7* expression was hormonally regulated, with transcript abundance markedly downregulated following MeJA treatment (Figure 4c). This regulatory pattern contrasts with the established expression profiles of both artemisinin biosynthetic enzymes and their positive regulatory transcription factors (Chen et al. 2016; Fu et al. 2021; Liu et al. 2023; Lu et al. 2013; Ma et al. 2018, 2021). The expression dynamics of *AaMYB7* strikingly mirror those of the previously characterised negative regulator AaMYB15, an R2R3‐MYB transcription factor (TF) reported to suppress artemisinin biosynthesis in *A. annua* (Wu et al. 2021). The distinct regulatory mechanisms of AaMYB7 and AaMYB15 are underscored by their structural divergence, particularly the absence of an EAR motif in AaMYB15. Our analysis revealed distinct patterns in jasmonate accumulations and the expressions of key biosynthetic genes across different leaf developmental stages. Notably, the levels of JA, jasmonoyl‐L‐isoleucine (JA‐Ile) and methyl jasmonate (MeJA) exhibited a progressive decline as leaves matured (Figure S6a–c). The expression levels of genes encoding core enzymes in the JA biosynthetic pathway were significantly decreased. Specifically, the expression of *ALLENE OXIDE CYCLASE (AOC)* and *12‐OXOPHYTODIENOATE REDUCTASE 3* (*OPR3*) decreased markedly with advancing leaf development (Figure S6d,e), consistent with the observed decline in JA, JA‐Ile and MeJA pools. However, in contrast to *AOC* and *OPR3*, the expression levels of *ALLENE OXIDE SYNTHASE* (*AOS*) remained relatively stable and showed no statistically significant changes across the developmental stages examined (Figure S6f). This reciprocal relationship aligns with our earlier observation that JA signalling may transcriptionally suppress *AaMYB7* expression, suggesting that JA‐mediated suppression of *AaMYB7* during early leaf expansion permits artemisinin biosynthesis, while developmental JA decay in aging leaves licences AaMYB7‐mediated transcriptional shutdown of the pathway. Of course, elevated DHAA levels also exert feedback inhibition on key biosynthetic enzyme genes. Our results show that the discovery of AaMYB7 as a negative regulator introduces a critical balancing mechanism in artemisinin regulation.

### 3.3 AabHLH93–AaWRKY9 Synergistically Activate Artemisinin Biosynthetic Promoters, While AaMYB7 Antagonises Their Combinatorial Activation

**FIGURE 6:**
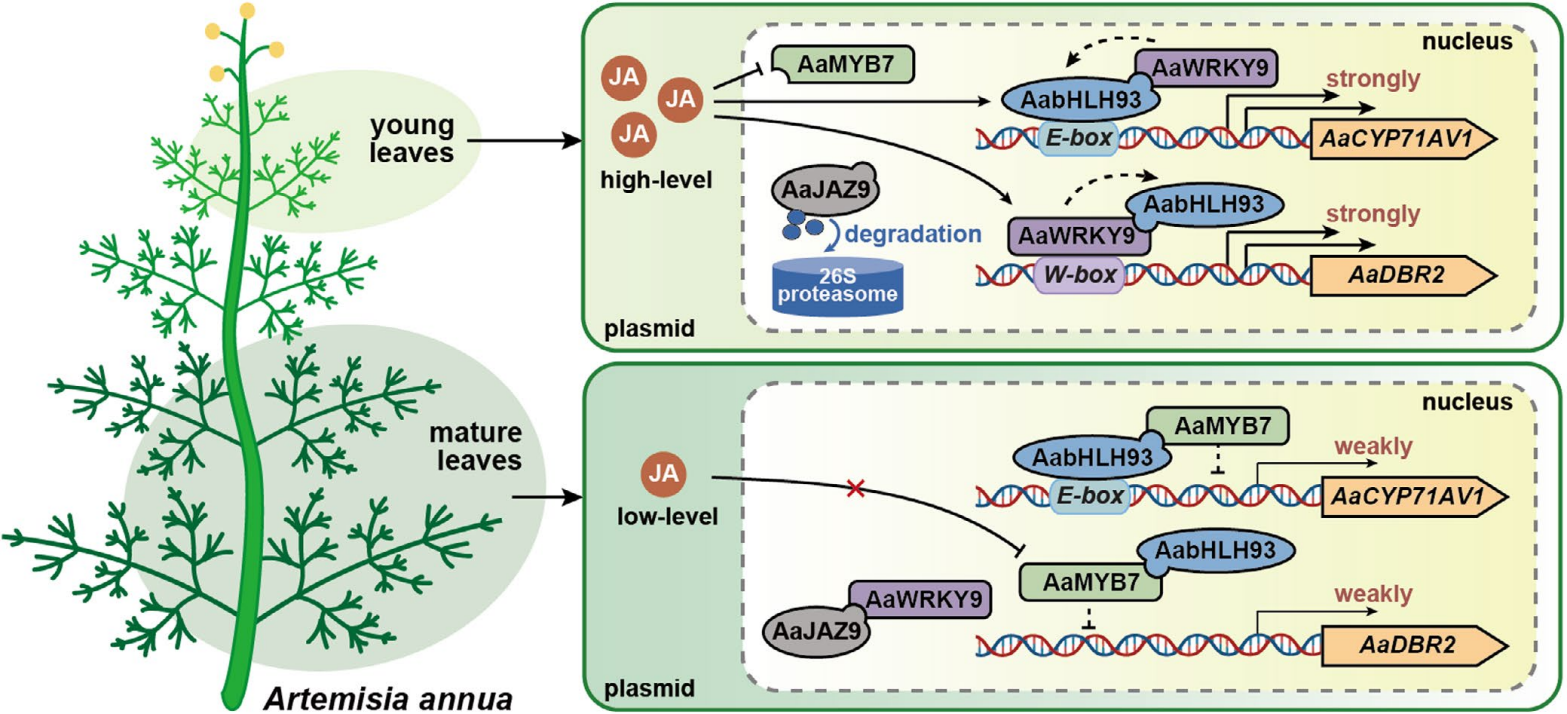
A working model showing that AabHLH93–AaWRKY9 and AabHLH93–AaMYB7 complexes regulate artemisinin biosynthesis. In young leaves, elevated JA levels repress the expression of *AaMYB7* and promote the degradation of AaJAZ9 via the 26S proteasome. The absence of AaMYB7 and AaJAZ9 allows the formation of the AabHLH93–AaWRKY9 activation complex. This complex robustly activates the expression of artemisinin biosynthetic genes, leading to high artemisinin production. In mature leaves, age‐dependent depletion of JA leads to the upregulation of *AaMYB7* and accumulation of AaJAZ9. AaMYB7 directly binds to AabHLH93 via its C‐terminal domain, displacing AaWRKY9 and occluding the transactivation interface. This interaction destabilises the AabHLH93–AaWRKY9 complex, weakening the activation of artemisinin biosynthetic genes.

Dual‐LUC assays revealed that the interaction between AabHLH93 and AaWRKY9 synergistically enhances the activation of artemisinin biosynthesis (Figure 2j), demonstrating that AabHLH93 and AaWRKY9 form a co‐regulatory module that amplifies transcriptional activation of artemisinin biosynthetic genes through both direct promoter binding and mutual reinforcement. Besides, AaWRKY9 not only activates downstream biosynthetic genes but also upregulates *AabHLH93* expression, thereby creating a self‐reinforcing cascade to maximise pathway flux under favourable conditions. And AaJAZ9 significantly attenuates AaWRKY9‐driven transcriptional activation of the *AabHLH93* promoter (Figure S3). This coordinated regulatory mechanism enables rapid responses to environmental signals to promote artemisinin biosynthesis. However, the negative regulator AaMYB7 interacting with AabHLH93 imposes a transcriptional brake on AabHLH93‐driven activation of artemisinin biosynthetic promoters and also directly antagonises the transcriptional activation capacity of the AabHLH93–AaWRKY9 complex through recruiting corepressor (Figures 3 and 5a,g,h,i,j). Interestingly, Y2H assays and the docking models of AaWRKY9–AaJAZ9, AaWRKY9–AabHLH93 and AabHLH93–AaMYB7 predicted by AlphaFold3 demonstrate that AaJAZ9 and AabHLH93 bind to the C‐terminal domain of AaWRKY9, while both AaWRKY9 and AaMYB7 interact with the C‐terminal domain of AabHLH93 (Figure 5b–f). These results demonstrated that AaMYB7 functions as a competitive inhibitor, disrupting the activity and stability of the AaWRKY9–AabHLH93 activation complex. The intricate regulatory network controlling artemisinin biosynthesis in *A. annua* involves a dynamic interplay of transcriptional activators and repressors, as revealed by the synergistic and antagonistic interactions between AaWRKY9, AabHLH93, AaMYB7 and AaJAZ9. The ontogenetic decline in JA levels from mature to senescent leaves establishes a spatiotemporal control framework for artemisinin production. Combined, these results elevate JA repress *AaMYB7* expression and promote 26S proteasome‐mediated degradation of AaJAZ9, while stabilising the AabHLH93–AaWRKY9 complex, enabling robust activation of artemisinin biosynthetic genes. Conversely, age‐dependent JA depletion permits *AaMYB7* upregulation and AaJAZ9 accumulation; AaMYB7 directly binds AabHLH93 via its C‐terminal domain, sterically displacing AaWRKY9 while occluding the transactivation interface, to destabilise the AabHLH93–AaWRKY9 complex, thereby weakening artemisinin biosynthesis (Figure 6).

## 4 Methods and Materials

### 4.1 Plant Material and Treatments

Seeds of *A. annua* (cv. Huhao1), originally collected from Chongqing, China, were subjected to multi‐year selective breeding under Shanghai agroclimatic conditions. Both *A. annua* and *N. benthamiana* plants were maintained in controlled greenhouse environments with 24°C diurnal cycles and a 16/8 h (light/dark) photoperiod to synchronise growth phases. Light and MeJA treatments were carried out as described previously (Fu et al. 2021).

### 4.2 Y2H and Luciferase Complementation Assays

The cDNA library derived from the leaves and meristem mRNAs of *A. annua* was generated by OE BioTech (Shanghai, China) for interaction screening (Ma et al. 2018). The coding sequences of *AaWRKY9* and *AabHLH93* were individually cloned into the pGBKT7 vector as the bait. Y2H screening assays were conducted following the manufacturer's protocol for the Matchmaker Gold Y2H system (Takara, Japan) (Ma et al. 2018). For yeast two‐hybrid analysis, *AabHLH93* and *AaM*YB7 were cloned into the pGADT7 vector and co‐transformed with the bait constructs into AH109 yeast cells. Following selection on SD/‐Leu/‐Trp plates, positive colonies were subsequently replica‐plated onto both SD/‐Leu/‐Trp/‐His and SD/‐Leu/‐Trp/‐His/‐Ade media. Protein interaction strength was assessed through colony growth monitoring over a 72‐h incubation period. The primers are listed in Table S1.

Luciferase complementation assays were performed as previously established (Fu et al. 2025). The *AabHLH93* was fused to the N‐terminal luciferase fragment in the pCAMBIA1300‐nLUC vector, whereas both *AaWRKY9* and *AaM*YB7 were respectively ligated into the C‐terminal counterpart (pCAMBIA1300‐cLUC). Recombinant constructs and empty vector controls were respectively introduced into *Agrobacterium* GV3101 cells harbouring the p19. These combinations of recombinant plasmids were infiltrated into *N. benthamiana* leaves, respectively (Voinnet et al. 2003). The luminescent reactions were processed using a Stable‐Lite Luciferase Assay System (Vazyme, China), followed by quantitative detection through a ChemiDoc MP Imaging System (Bio‐Rad, USA) for interaction strength assessment.

### 4.3 BiFC and Co‐IP Assays

For BiFC assays, the open reading frame (ORF) of *AabHLH93* was fused to the N‐terminal luciferase fragment, and the full‐length coding regions of both *AaWRKY9* and *AaM*YB7 were inserted into the pxy106 vector. The recombinant plasmids and the corresponding empty vectors were respectively introduced into GV3101. Following the transient transformation protocol (Voinnet et al. 2003), pairwise combinations of these constructs were co‐delivered into *N. benthamiana* leaves. YFP‐specific emission signals were captured under a Leica TCS SP5‐II confocal laser microscope.

For CoIP assays, the open reading frame of *AabHLH93* and *AaMYB7* was inserted into the pHB‐YFP vector. The *AaWRKY9* coding sequence was fused to an N‐terminal Flag tag, while *AabHLH93* was cloned into the pHB‐FLAG vector. The recombinant plasmids (pHB‐*AabHLH93*‐YFP paired with pHB‐Flag‐*AaWRKY9*, and pHB‐*AaMYB7*‐YFP combined with pHB‐*AabHLH93*‐Flag) alongside empty vector controls were individually introduced into GV3101. The *N. benthamiana* leaves were co‐infiltrated with bacterial suspensions harbouring plasmid pairs and the p19. After 48 h of incubation, leaf tissues were harvested and flash‐frozen in liquid nitrogen prior to pulverisation. CoIP procedures followed established protocols (Ma et al. 2018). Briefly, total protein extracts from infiltrated leaves were then mixed with 25 μL of anti‐GFP nanobody agarose beads (Alpalifebio, China) and incubated under identical conditions. Immunoprecipitated complexes were analysed by immunoblotting using anti‐GFP (Abmart, China) and anti‐Flag monoclonal antibodies (Sigma‐Aldrich, USA).

### 4.4 Dual‐LUC Assays

The transcription factor genes *AaWRKY9*, *AabHLH93* and *AaMYB7* were inserted into the pHB vector. Correspondingly, promoter regions of *AaADS*, *AaCYP71AV1*, *AaDBR2*, *AaALDH1* and *AaAabHLH93* were ligated into the pGREEN0800‐LUC reporter vector. The empty pHB vector served as the effector negative control. Reporter constructs were co‐transformed with the helper plasmid pSoup19 into *A. tumefaciens* GV3101, while effector constructs were introduced into GV3101 without additional plasmids. Bacterial suspensions containing these constructs were agroinfiltrated into *N. benthamiana* leaves. Three independent biological replicates were performed for all effector‐reporter combinations.

### 4.5 Y1H and Electrophoretic Mobility Shift Assays

The coding sequence of *AabHLH93* was PCR‐amplified and directionally cloned into the pB42AD bait vector. To assess transcriptional activation, trimerized cis‐elements containing G‐box and E‐box motifs derived from the promoters of artemisinin biosynthetic genes were synthesised and ligated into the pLacZ reporter vector. A synthetic construct harbouring three concatenated G‐box/E‐box motifs served as a composite regulatory element control. Yeast strain EGY48 was transformed with recombinant pB42AD and pLacZ constructs, while parallel transformations using the empty pB42AD vector paired with cis‐element constructs provided negative controls. Transformants were selected on SD/‐Trp/‐Ura agar plates and subsequently subjected to β‐galactosidase activity assays by replica plating onto X‐gal‐supplemented induction medium, followed by 48‐h incubation at 30°C for chromogenic development.

The *AabHLH93* ORF was subcloned into the pCold‐TF expression vector (Takara, Japan) featuring a cold‐inducible expression system. Competent *Escherichia coli* Rosetta (DE3) cells (Weidi Biotech, China) were transformed with either recombinant or empty vectors for comparative analysis. Recombinant His‐TF fusion proteins were expressed through a 14‐h induction at 16°C with 200 μM IPTG (Isopropyl‐D‐thiogalatopyranoside), followed by affinity purification using Ni‐NTA agarose resin (Invitrogen, USA) under native conditions. For DNA‐protein interaction analysis, electrophoretic mobility shift assays were conducted employing the LightShift Chemiluminescent EMSA Platform (Thermo Fisher Scientific, USA). The wild‐type E‐box3 and the mutant E‐box3 were synthesised as biotinylated probes (Sangon Biotech, China).

## 5 Accession Numbers

The sequence data mentioned have been deposited in the following databases: TAIR and NCBI databases. The accession numbers are as follows: *AaWRKY9* (PV878106), *AabHLH93* (PV878107), *AaMYB7* (PV878108), *proADS* (DQ448294), *proCYP71AV1* (FJ870128), *proDBR2* (KC118523.1) and *proALDH1* (KC118525.1).

## Author Contributions

The research was designed by K.T. and X.F. Experimental work was conducted by X.F., H.Z., J.Z. and Y.W. Data analysis was performed by J.Z., S.L., M.Y., P.L., L.L. and X.S. Manuscript writing was supported by X.F. and H.Z.

## Conflicts of Interest

The authors declare no conflicts of interest.

## Supporting information


**Figure S1:** Phylogenetic analysis and alignment of the protein sequences of AabHLH93 and related proteins.
**Figure S2:** Subcellular localization of AabHLH93.
**Figure S3:** Dual‐LUC assay showing that AaWRKY9 activates the expression of *AabHLH93*.
**Figure S4:** Phylogenetic analysis and alignment of the protein sequences of AaMYB7 and related proteins.
**Figure S5:** Subcellular localization of AaMYB7.
**Figure S6:** Endogenous jasmonate levels and biosynthetic gene expression dynamics across the leaves at different developmental stages in *Artemisia annua* .
**Figure S7:** The genetic transformation and PCR identification of *AabHLH93* transgenic *Artemisia annua* lines.
**Figure S8:** HPLC chromatograms of artemisinin.

**Table S1:** Primers used in this study.

## Data Availability

Data sharing not applicable to this article as no datasets were generated or analysed during the current study.
