# Supplementary material for: Jasmonate‐Activated AaWRKY9–AabHLH93/AabHLH93–AaMYB7 Complexes Balance Artemisinin Biosynthesis in Artemisia annua
Source: Plant Biotechnol J. 2025 Oct 28;24(3):1533–47. doi: 10.1111/pbi.70416 (PMC12946470; doi:10.1111/pbi.70416)
Supplement: Supplementary file 1 — Figure S1: Phylogenetic analysis and alignment of the protein sequences of AabHLH93 and related proteins. Figure S2: Subcellular localization of AabHLH93. Figure S3: Dual‐LUC assay showing that AaWRKY9 activates the expression of AabHLH93. Figure S4: Phylogenetic analysis and alignment of the protein sequences of AaMYB7 and related proteins. Figure S5: Subcellular localization of AaMYB7. Figure S6: Endogenous jasmonate levels and biosynthetic gene expression dynamics across the leaves at different developmental stages in Artemisia annua . Figure S7: The genetic transformation and PCR identification of AabHLH93 transgenic Artemisia annua lines. Figure S8: HPLC chromatograms of artemisinin. [file PBI-24-1533-s002.zip › pbi70416-sup-0001-FiguresS1-S8/pbi70416-sup-0001-FiguresS1-S8/pbi70416-sup-0002-FigureS1-S8@Supporting Information.pdf]

## Methods and materials

### RNA extraction and qRT-PCR

The different tissues and the leaves at various developmental stages, including young leaves, old leaves, buds, roots, stems, flowers and trichomes, as well as foliar tissues at distinct developmental phases, were isolated from 5-month-old *A. annua*. RNA extraction was performed with the RNAprep Pure Plant Kit (Tiangen, Beijing, China) following established protocols (Ma et al., 2018). First-strand cDNA for qRT-PCR was achieved with the total RNA using the PrimeScript™ RT Master Mix (Takara, Shiga, Japan), followed by SYBR Green-based quantitative PCR amplification using the SuperReal PreMix Plus kit (Tiangen, Beijing, China). Gene-specific primers were validated with *A. annua β-ACTIN* as an endogenous normalization control. qRT-PCR assays were performed as previously described (Fu et al., 2021). Three biological repeats were measured for each sample. Evolutionary relationships were inferred using MEGA11 for phylogenetic tree reconstruction, while DNAMAN facilitated comprehensive multiple sequence alignments to assess nucleotide or protein homology patterns. All the primers used in this study are listed in Table S1.

### Subcellular localization

To determine the subcellular localization of AabHLH93 and AaMYB7 protein, the yellow fluorescent protein (YFP) was fused to the C-terminal domain of *AabHLH93* and *AaMYB7* under the control of the CaMV35S promoter. The recombinant plasmids and the empty vector as the control were respectively transferred into GV3101 harboring the p19. The strains GV3101 harboring recombinant plasmids and the empty vector were respectively co-transformed into *N. benthamiana* leaves. YFP signals was observed by confocal laser microscopy (Leica TCS SP5-II) after 48 hours incubation.

### Molecular cloning of promoter and promoter-GUS fusions in transgenic *A. annua*

The promoter sequences of *AabHLH93* and *AaMYB7* were predicted using the *A. annua* reference genome (Shen et al., 2018). The predicted promoter fragments of *AabHLH93* and *AaMYB7* were cloned with specific primers. The promoter fragments were inserted

into the *BamH*I and *Nco*I sites of pCAMBIA1391, respectively. The recombinant plasmids were transformed into *A. tumefaciens* strain EHA105 for the *A. annua* plant transformation (Jing et al., 2009). The GUS staining was performed as previously described (Ma et al., 2018).

**Construction and transformation of *A. annua***

The full-length coding regions of *AabHLH93* and *AaMYB7* were respectively cloned into the pHB vector. To construct the antisense lines, the 300 bp non-conservative domain coding sequence of *AabHLH93* and *AaMYB7* were cloned into pHellsgate12 vector. The constructs and pHB vector were transferred into strain EHA105 for the *A. annua* plant transformation (Jing et al., 2009)

**Measurement of artemisinin, DHAA and AA contents**

Fresh leaves from overexpression, antisense, the transgenic lines transformed with the empty vector and wild type plants were collected, dried at 50°C for 48 h, and ground to powder. 0.1 g/sample was extracted twice with 1 ml methanol. After centrifuging at 12000 rpm for 10 min, the supernatant was collected for the measurement of artemisinin, DHAA and AA content, as described previously (Jing et al., 2009). All the assays were repeated for three times.

**Protein-protein interaction prediction using AlphaFold3**

The structural prediction of protein-protein interactions was performed using AlphaFold3 (https://alphafoldserver.com/). Statistical analysis of interface residues was performed using PyMOL (v3.1).

**Quantitative analysis of endogenous jasmonic acid (JA), methyl jasmonate (MeJA), and jasmonoyl-isoleucine (JA-Ile) contents**

The quantitative analysis of JA, MeJA and JA-Ile were performed with modifications according to the previously published method (Pan et al., 2010). Fresh plant tissues were ground to a fine powder in liquid nitrogen using a pre-chilled mortar and pestle. For each

sample, 50 mg of powdered tissue was weighed and transferred into a 2 ml screw-cap tube and kept in liquid nitrogen. A 50 µl aliquot of the internal standard working solution (50 µg ml$^{-1}$) was added to each tube, followed by 500 µl of extraction solvent consisting of 2-propanol: distilled $H_2O$: concentrated HCl (2: 1: 0.002, v/v/v). For samples exceeding 50 mg, the volume of extraction solvent was adjusted to maintain a tissue-to-solvent ratio of 1:10 (mg: µl). The samples were shaken at 100 rpm for 30 min at 4 °C, followed by the addition of 1 ml dichloromethane. After shaking for an additional 30 min at 4 °C, the samples were centrifuged at 13,000 ×g for 5 min at 4 °C. After centrifugation, the lower organic phase (\~900 µl) was carefully collected using a Pasteur pipette and transferred to a screw-cap vial. The extract was concentrated under a gentle stream of nitrogen gas, avoiding complete dryness, and reconstituted in 100 µl methanol. A 50 µl aliquot was injected into a reverse-phase $C_{18}$ Gemini HPLC column for HPLC-ESI-MS/MS analysis. Hormone quantification was performed using an ultra-performance liquid chromatography system (ExionLC™ AD) coupled with a tandem mass spectrometer (QTRAP® 6500+). Chromatographic separation was achieved using a Waters ACQUITY UPLC HSS T3 C18 column (1.8 µm, 100 mm × 2.1 mm i.d.) with a mobile phase consisting of solvent A (ultrapure water with 0.04% acetic acid) and solvent B (acetonitrile with 0.04% acetic acid). Mass spectrometry was conducted using an electrospray ionization (ESI) source in both positive (5500 V) and negative (−4500 V) ion modes at a source temperature of 550 °C. Curtain gas was set to 35 psi.

## References

Fu, X.Q., Peng, B.W., Hassani, D., Xie, L.H., Liu, H., Li, Y.P., Chen, T.T., Liu, P., Tang, Y.L., Li, L., Zhao, J.Y., Sun, X.F. and Tang, K.X. (2021) AaWRKY9 contributes to light- and jasmonate-mediated to regulate the biosynthesis of artemisinin in *Artemisia annua*. *New Phytol* **231**, 1858-1874.

Jing, F.X., Zhang, L., Li, M.Y., Tang, Y.L., Wang, YY.., Wang, Y., Wang, Q., Pan, Q.F., Wang, G.F. and Tang, K.X. Abscisic Acid (ABA) treatment increases artemisinin content in Artemisia annua by enhancing the expression of genes in artemisinin biosynthetic pathway, *Biologia*, 2009, 64, 319–323.

Ma, Y.N., Xu, D.B., Li, L., Zhang, F., Fu, X.Q., Shen, Q., Lyu, X.Y., Wu, Z.K., Pan, Q.F., Shi, P., Hao, X.L., Yan, T.X., Chen, M.H., Liu, P., He, Q., Xie, L.H., Zhong, Y.J., Tang, Y.L., Zhao, J.Y., Zhang, L.D., Sun, X.F. and Tang, K.X. (2018) Jasmonate promotes artemisinin biosynthesis by

activating the TCP14-ORA complex in *Artemisia annua*. *Sci Adv* **4**, eaas9357.
Pan, X.Q., Welti, R., Wang, X.M. Quantitative analysis of major plant hormones in crude plant extracts by high-performance liquid chromatography-mass spectrometry. *Nat Protoc*, 2010, 5(6), 986-92.
Shen, Q., Zhang, L.D., Liao, Z.H., Wang, S.Y., Yan, T.X., Shi, P., Liu, M., Fu, X.Q., et al. The genome of artemisia annua provides insight into the evolution of Asteraceae family and artemisinin biosynthesis. *Mol Plant*, 2018, Jun 4;11(6):776-788, doi: 10.1016/j.molp.2018.03.015.

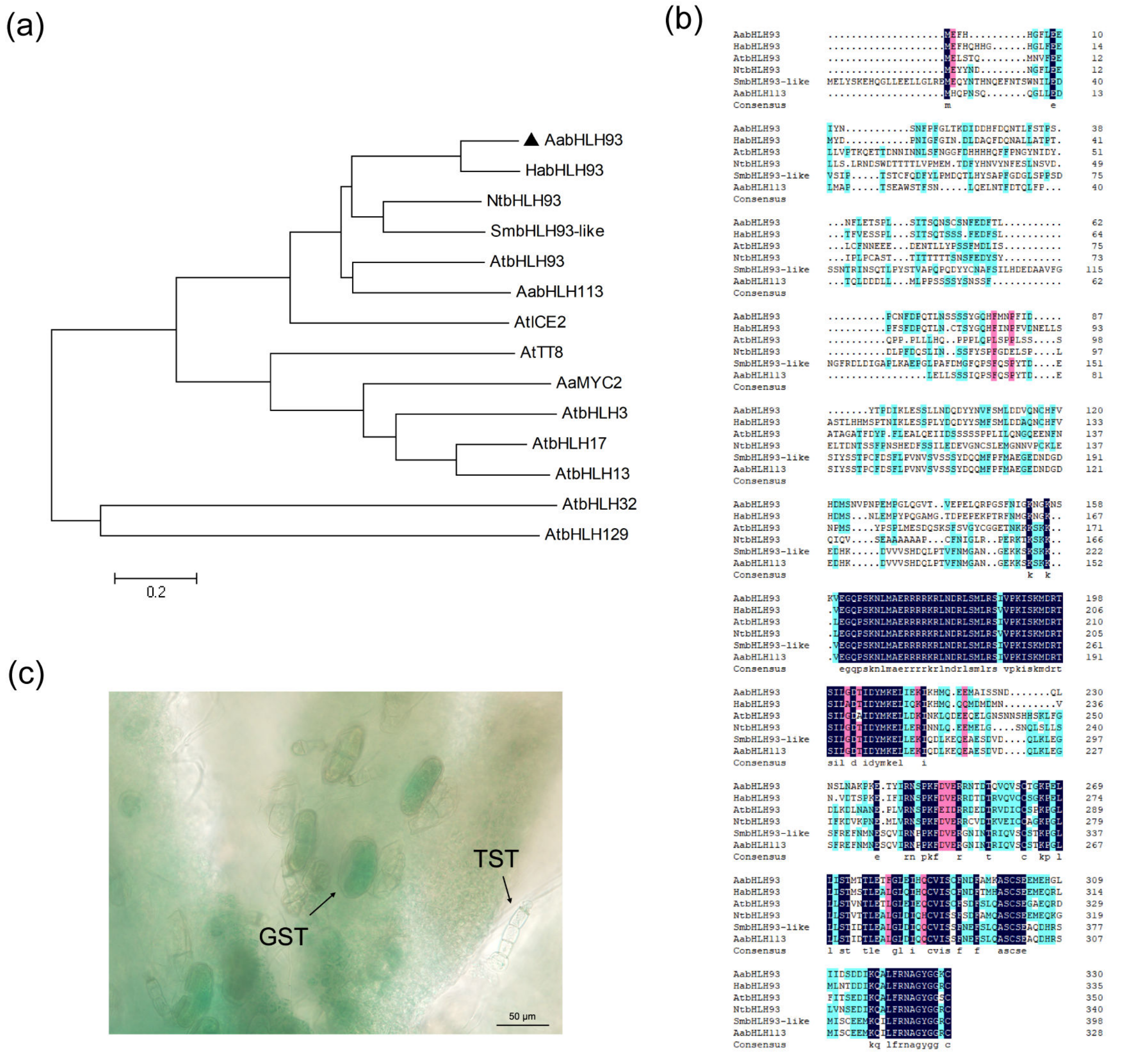


Figure S1 Phylogenetic analysis and alignment of the protein sequences of AabHLH93 and related proteins. (a) The neighbor-joining phylogenetic tree was constructed using MEGA. Bootstrap values indicate the percentage of 1000 replicates. AabHLH93 is marked with a black triangle. (b) Alignment of the amino acid sequences of AabHLH93 and other bHLH proteins. Identical amino acids are shaded in black. The accession numbers of the sequences shown are as follows: AabHLH113 (UYZ96440.1) and AaMYC2 (AKO62850.1) from *A. annua*; HabHLH93 (XP_021987100.1) from *Helianthus annuus*; NtbHLH93 (XP_016487716.1) from *Nicotiana tabacum*; SmbHLH93-like (XP_057794256.1) from *Salvia miltiorrhiza*; AtbHLH93 (AT5G65640.1), AtICE2 (AT1G12860.1), AtbHLH17 (AT2G46510.1), AtTT8 (AT4G09820.1), AtbHLH13 (AT1G01260.1), AtbHLH32 (AT3G25710.1), AtbHLH3 (AT4G16430.1) and AtbHLH129 (AT2G43140.2) from *Arabidopsis thaliana*. (c) GUS staining of the *proAabHLH93-GUS* transgenic *A. annua*. GST, glandular trichome; TST, T-shaped trichome. Bar=50 μm.

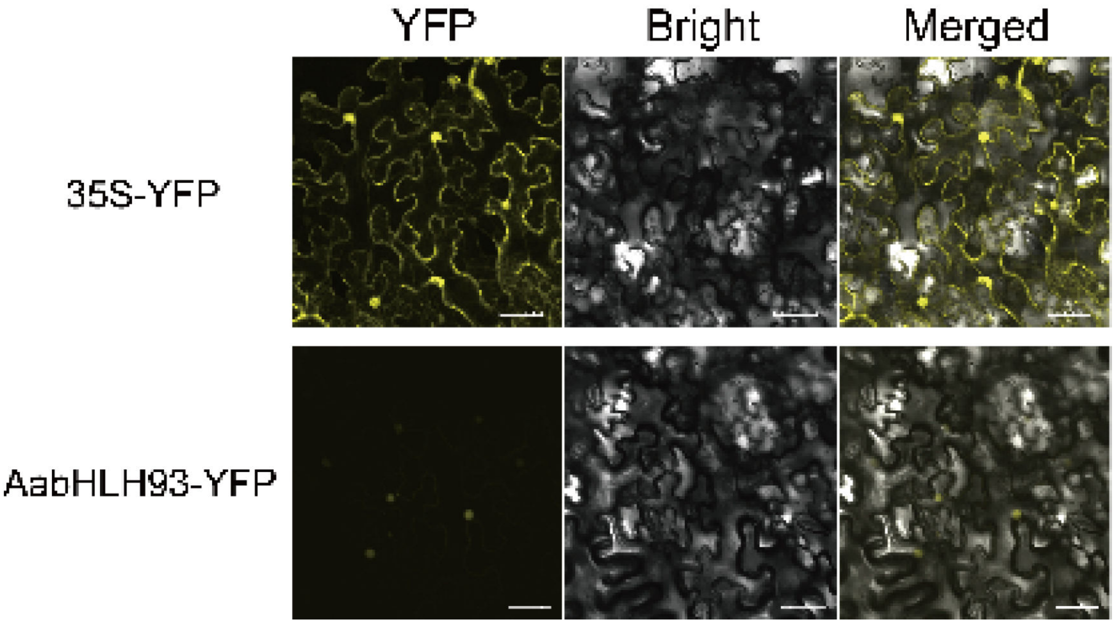


Figure S2 Subcellular localization of AabHLH93. YFP signal was used as the control. Bar=50μm.

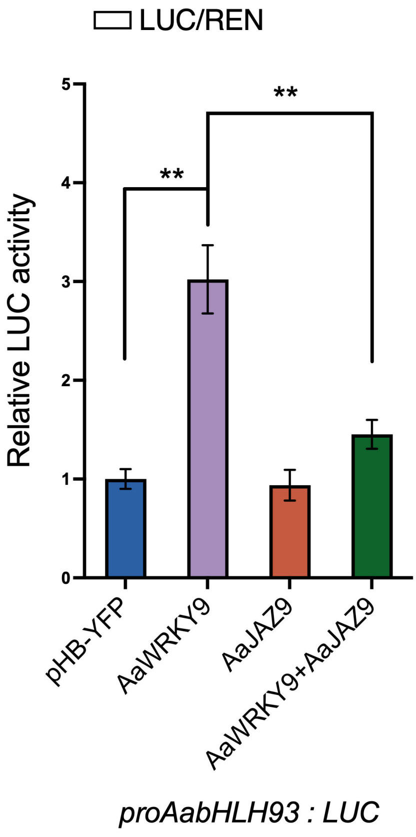


Figure S3 Dual-LUC assay showing that AaWRKY9 activates the expression of *AabHLH93*. AaJAZ9 protein represses the transcriptional activation function of AaWRKY9. The empty vector pHB-YFP was used as the control. The relative LUC activity of AaWRKY9 are comparisons relative to that of pHB-YFP. Asterisks denote a significant difference of AaWRKY9 relative to pHB-YFP as determined by a Student's *t*-test: **, P < 0.01.

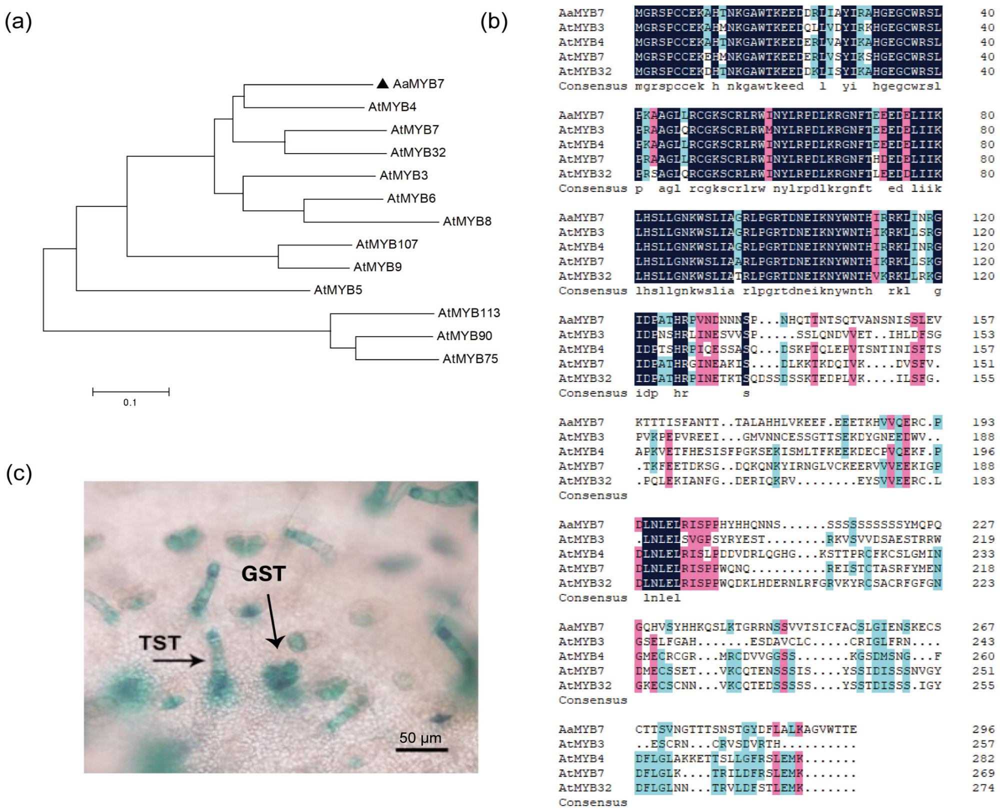


Figure S4 Phylogenetic analysis and alignment of the protein sequences of AaMYB7 and related proteins. (a) The neighbor-joining phylogenetic tree was constructed using MEGA. Bootstrap values indicate the percentage of 1000 replicates. AaMYB7 is marked with a black triangle. (b) Alignment of the amino acid sequences of AaMYB7 and other MYB proteins. Identical amino acids are shaded in black. The accession numbers of the sequences shown are as follows: AtMYB90 (AT1G66390.1), AtMYB113 (AT1G66370.1), AtMYB75 (AT1G56650.1), AtMYB3 (AT1G22640.1), AtMYB5 (AT3G13540.1), AtMYB4 (AT4G38620.1), AtMYB6 (AT4G09460.1), AtMYB7 (AT2G16720.1), AtMYB32 (AT4G34990.1), AtMYB107 (AT3G02940.1), AtMYB6 (AT4G09460.1), AtMYB8 (AT1G35515.1) and AtMYB9 (AT5G16770.1) from *Arabidopsis thaliana*. (c) GUS staining of the *proAaMYB7-GUS* transgenic *A. annua*. GST, glandular trichome; TST, T-shaped trichome. Bar=50 μm.

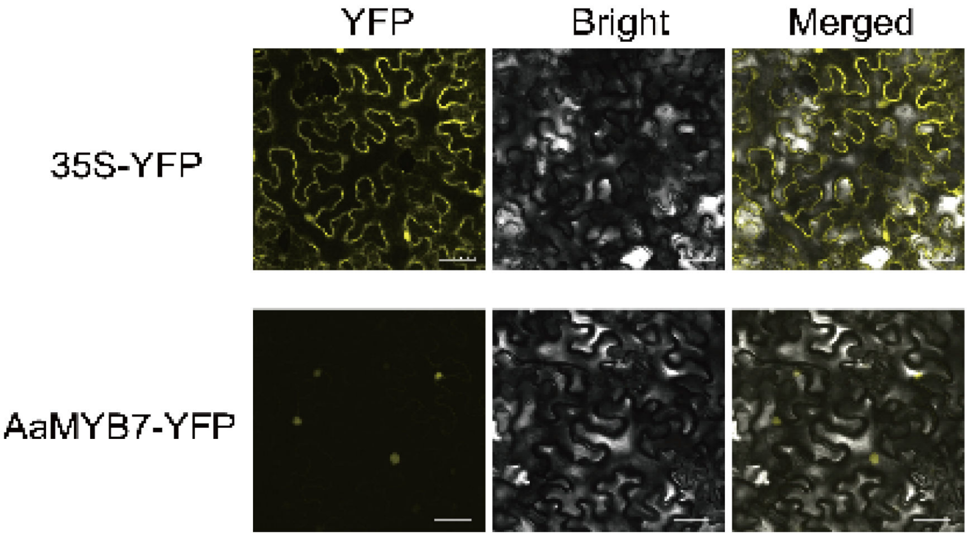


Figure S5 Subcellular localization of AaMYB7. YFP signal was used as the control. Bar=50μm.

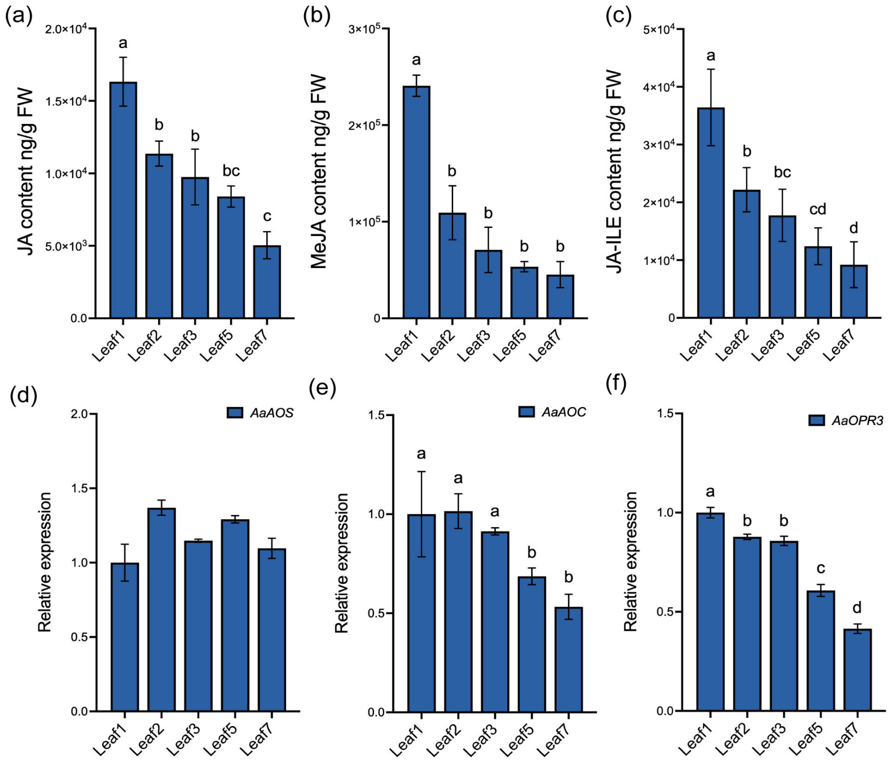


Figure S6 Endogenous jasmonate levels and biosynthetic gene expression dynamics across the leaves at different developmental stages in *A. annua*. Quantification of endogenous jasmonic acid (JA) (a), methyl jasmonate (MeJA) (b), and jasmonoyl-isoleucine (JA-Ile) (c)concentrations in leaves. (e-g)  Relative expression profiles of JA biosynthesis genes (*AaAOS*, *AaAOC* and *AaOPR3*). Transcript levels normalized to *ACTIN* reference gene. The values are presented as means ± s.d. (n = 3 independent experiments). Means were compared using one-way analysis of variance (ANOVA) followed by Tukey's multiple comparison tests.

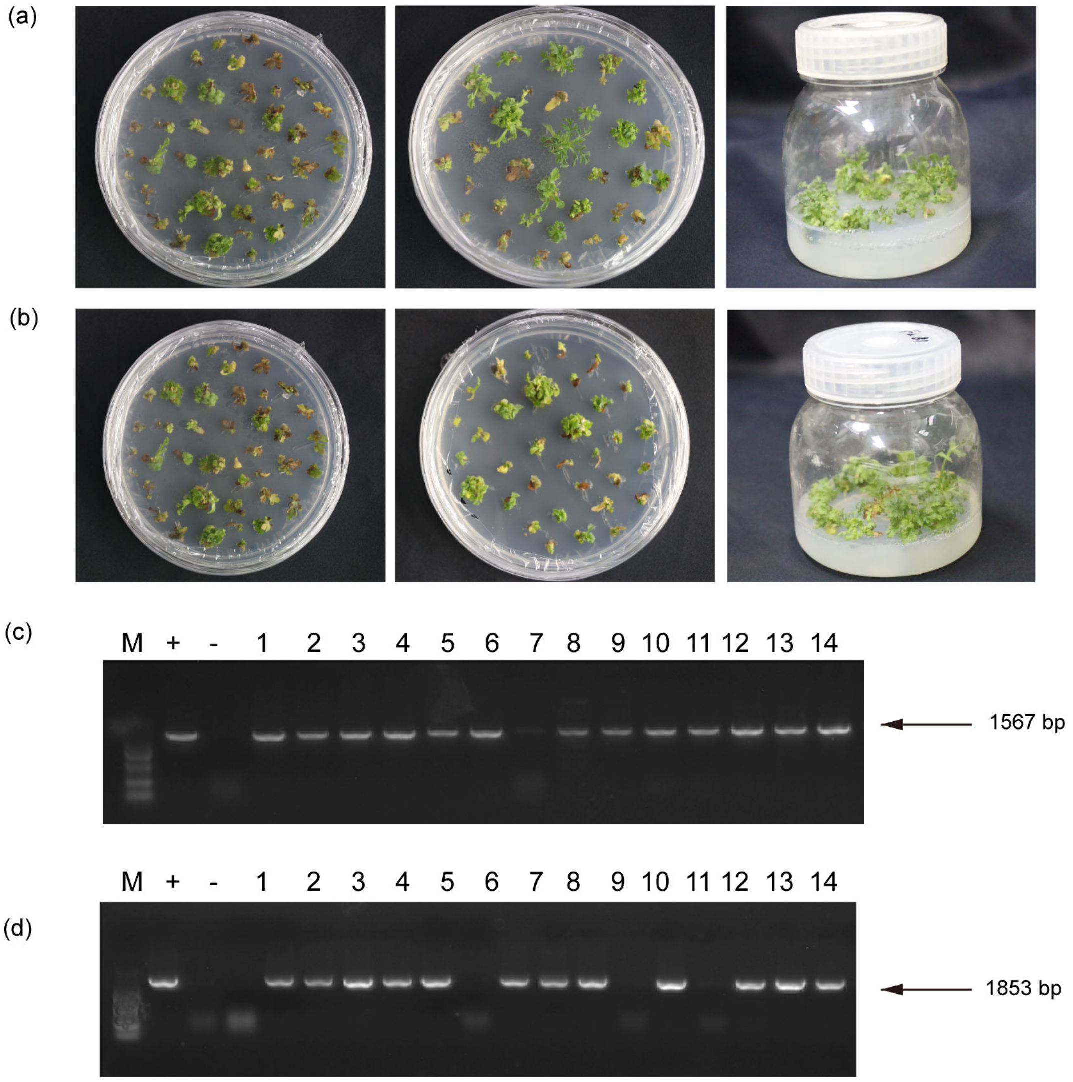


Figure S7 The genetic transformation and PCR identification of *AabHLH93* transgenic *A. annua* lines. (a) Representative images showing callus induction, shoot regeneration and root formation in *AabHLH93* overexpression transgenic lines. (b) Representative images showing callus induction, shoot regeneration and root formation in *AabHLH93*-RNAi transgenic lines. (c) PCR verification of *AabHLH93* overexpression transgenic lines. (d) PCR verification of *AabHLH93*-RNAi transgenic lines.

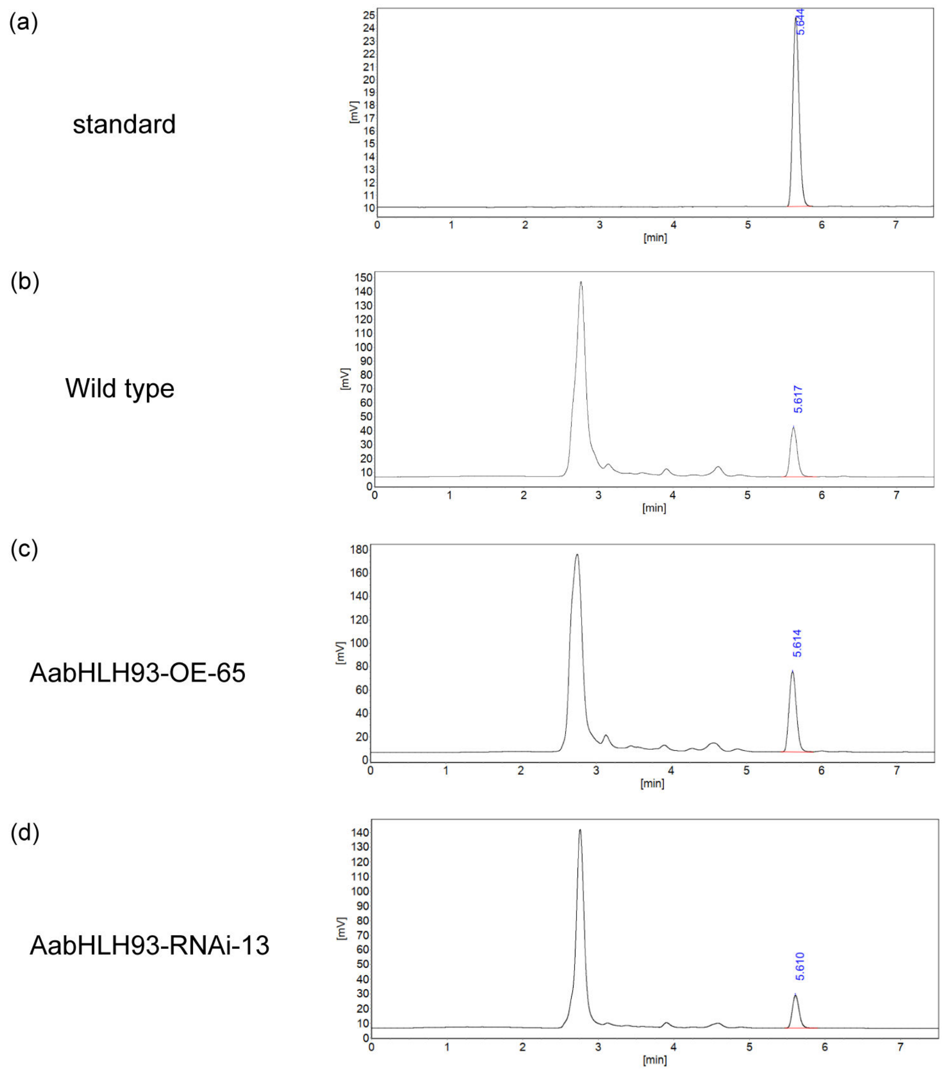


Figure S8 HPLC chromatograms of artemisinin. Shown are HPLC chromatograms of (a) the artemisinin standard (500 μg/mL), the extract solution of wild-type *A. annua* (b), *AabHLH93* overexpressing transgenic lines (c) and*AabHLH93*-RNAi transgenic lines (d).
