# Supplementary figures and images for: Jasmonate‐Activated AaWRKY9–AabHLH93/AabHLH93–AaMYB7 Complexes Balance Artemisinin Biosynthesis in Artemisia annua
Source: Plant Biotechnol J. 2025 Oct 28;24(3):1533–47. doi: 10.1111/pbi.70416 (PMC12946470; doi:10.1111/pbi.70416)

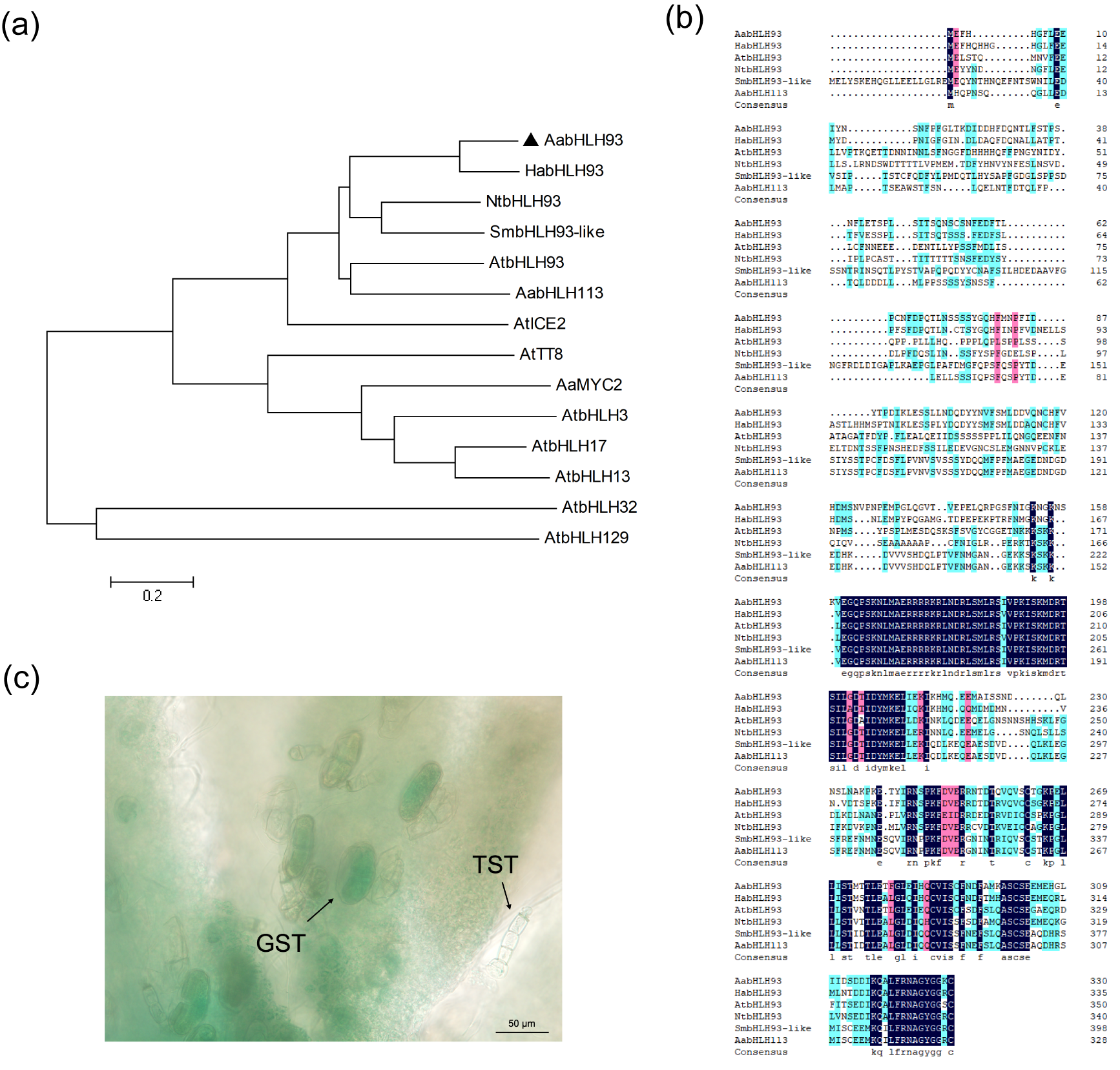

Supplement: Supplementary file 1 — Figure S1: Phylogenetic analysis and alignment of the protein sequences of AabHLH93 and related proteins. Figure S2: Subcellular localization of AabHLH93. Figure S3: Dual‐LUC assay showing that AaWRKY9 activates the expression of AabHLH93. Figure S4: Phylogenetic analysis and alignment of the protein sequences of AaMYB7 and related proteins. Figure S5: Subcellular localization of AaMYB7. Figure S6: Endogenous jasmonate levels and biosynthetic gene expression dynamics across the leaves at different developmental stages in Artemisia annua . Figure S7: The genetic transformation and PCR identification of AabHLH93 transgenic Artemisia annua lines. Figure S8: HPLC chromatograms of artemisinin. [file PBI-24-1533-s002.zip › pbi70416-sup-0001-FiguresS1-S8/pbi70416-sup-0001-FiguresS1-S8/pbi70416-sup-0001-FigureS1.tif]

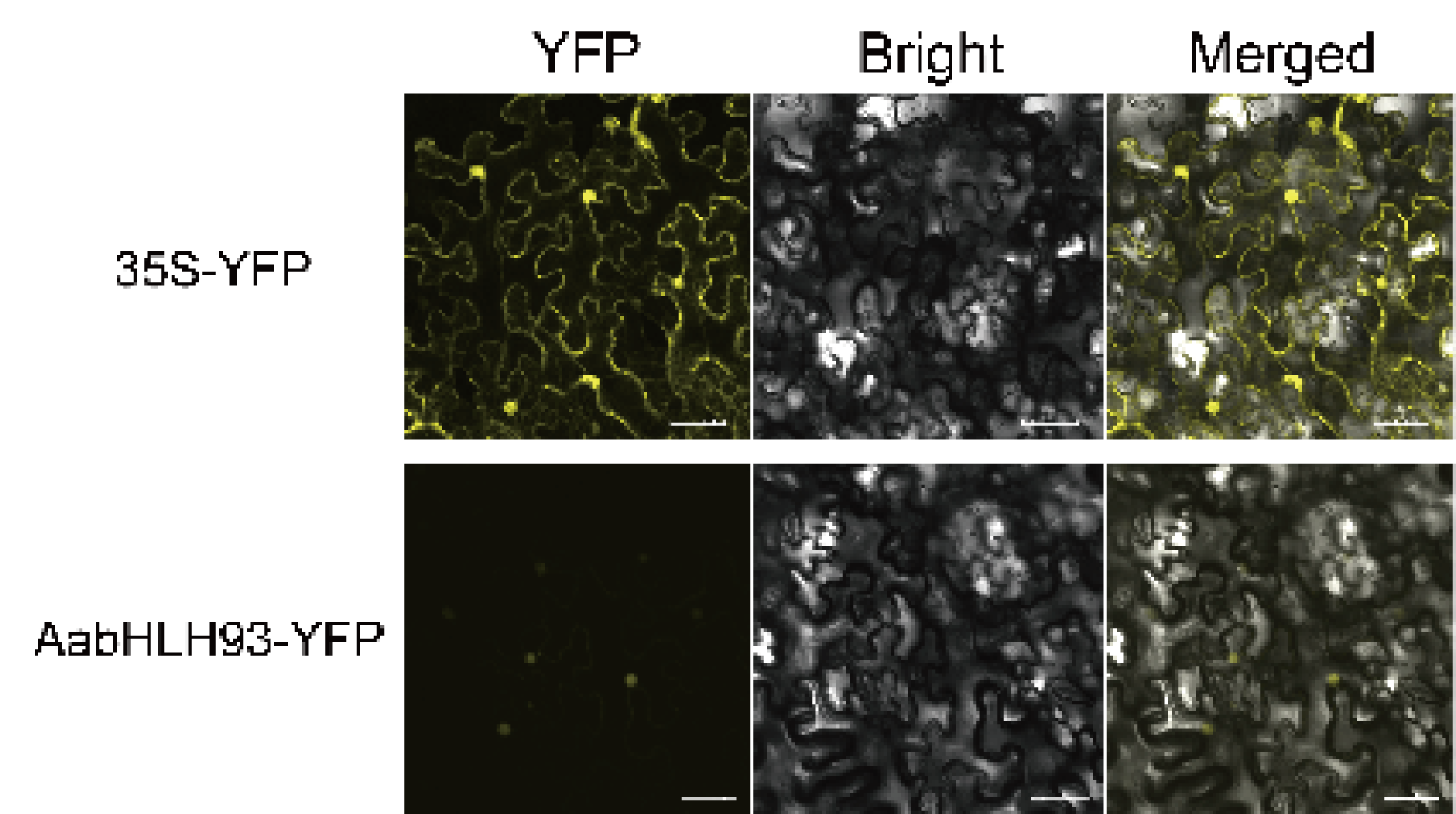

Supplement: Supplementary file 1 — Figure S1: Phylogenetic analysis and alignment of the protein sequences of AabHLH93 and related proteins. Figure S2: Subcellular localization of AabHLH93. Figure S3: Dual‐LUC assay showing that AaWRKY9 activates the expression of AabHLH93. Figure S4: Phylogenetic analysis and alignment of the protein sequences of AaMYB7 and related proteins. Figure S5: Subcellular localization of AaMYB7. Figure S6: Endogenous jasmonate levels and biosynthetic gene expression dynamics across the leaves at different developmental stages in Artemisia annua . Figure S7: The genetic transformation and PCR identification of AabHLH93 transgenic Artemisia annua lines. Figure S8: HPLC chromatograms of artemisinin. [file PBI-24-1533-s002.zip › pbi70416-sup-0001-FiguresS1-S8/pbi70416-sup-0001-FiguresS1-S8/pbi70416-sup-0003-FigureS2.tif]

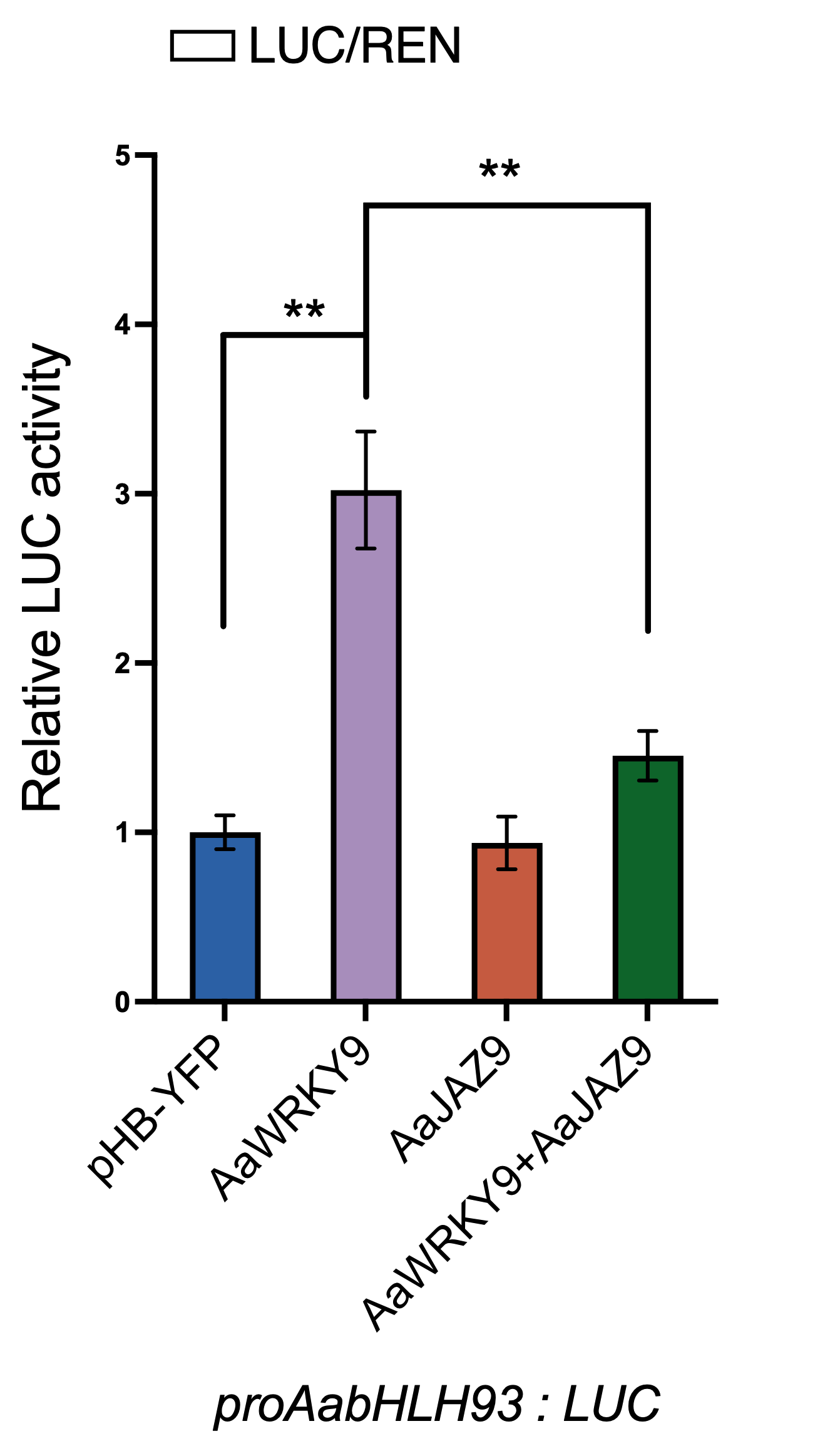

Supplement: Supplementary file 1 — Figure S1: Phylogenetic analysis and alignment of the protein sequences of AabHLH93 and related proteins. Figure S2: Subcellular localization of AabHLH93. Figure S3: Dual‐LUC assay showing that AaWRKY9 activates the expression of AabHLH93. Figure S4: Phylogenetic analysis and alignment of the protein sequences of AaMYB7 and related proteins. Figure S5: Subcellular localization of AaMYB7. Figure S6: Endogenous jasmonate levels and biosynthetic gene expression dynamics across the leaves at different developmental stages in Artemisia annua . Figure S7: The genetic transformation and PCR identification of AabHLH93 transgenic Artemisia annua lines. Figure S8: HPLC chromatograms of artemisinin. [file PBI-24-1533-s002.zip › pbi70416-sup-0001-FiguresS1-S8/pbi70416-sup-0001-FiguresS1-S8/pbi70416-sup-0004-FigureS3.tiff]

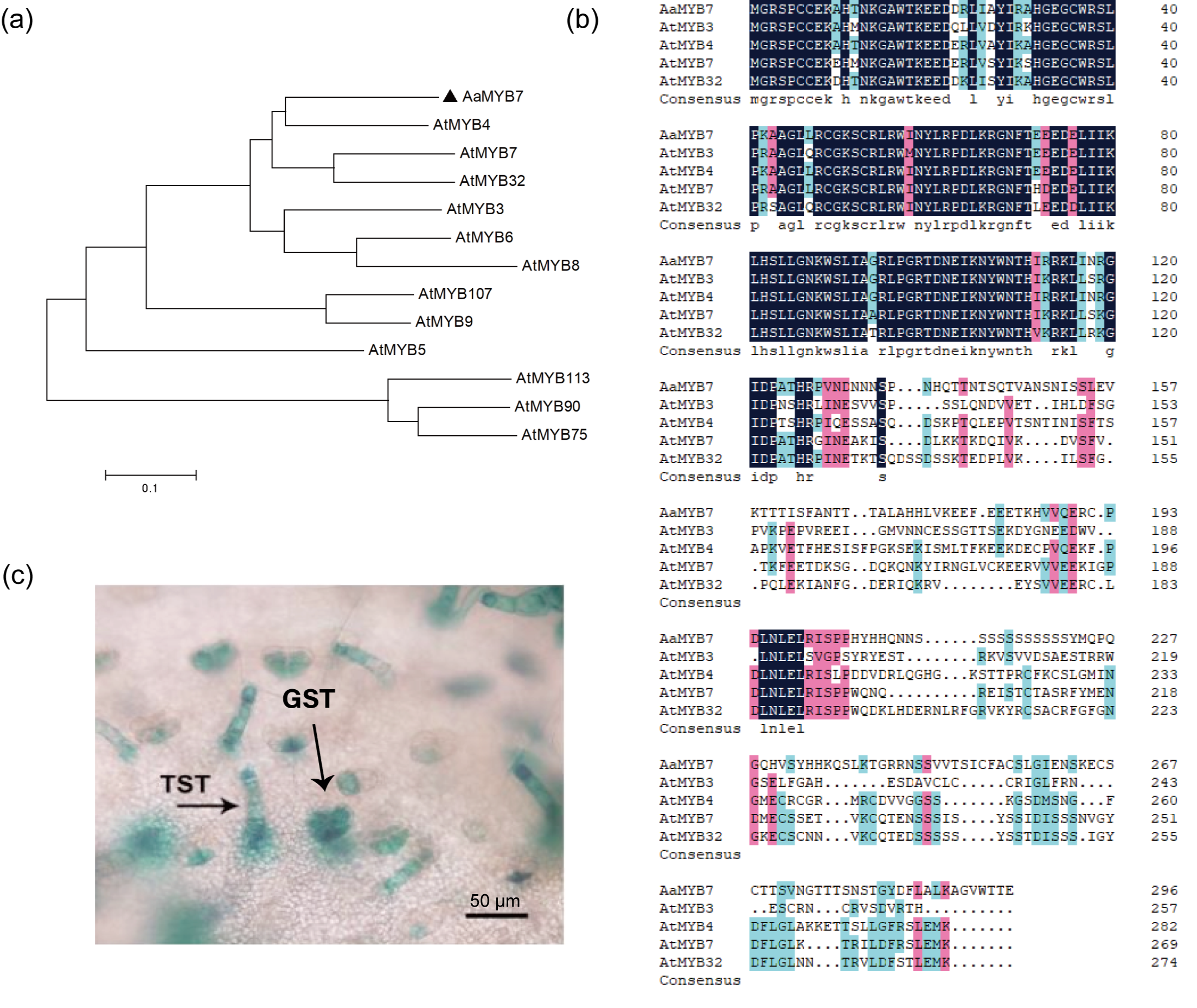

Supplement: Supplementary file 1 — Figure S1: Phylogenetic analysis and alignment of the protein sequences of AabHLH93 and related proteins. Figure S2: Subcellular localization of AabHLH93. Figure S3: Dual‐LUC assay showing that AaWRKY9 activates the expression of AabHLH93. Figure S4: Phylogenetic analysis and alignment of the protein sequences of AaMYB7 and related proteins. Figure S5: Subcellular localization of AaMYB7. Figure S6: Endogenous jasmonate levels and biosynthetic gene expression dynamics across the leaves at different developmental stages in Artemisia annua . Figure S7: The genetic transformation and PCR identification of AabHLH93 transgenic Artemisia annua lines. Figure S8: HPLC chromatograms of artemisinin. [file PBI-24-1533-s002.zip › pbi70416-sup-0001-FiguresS1-S8/pbi70416-sup-0001-FiguresS1-S8/pbi70416-sup-0005-FigureS4.tif]

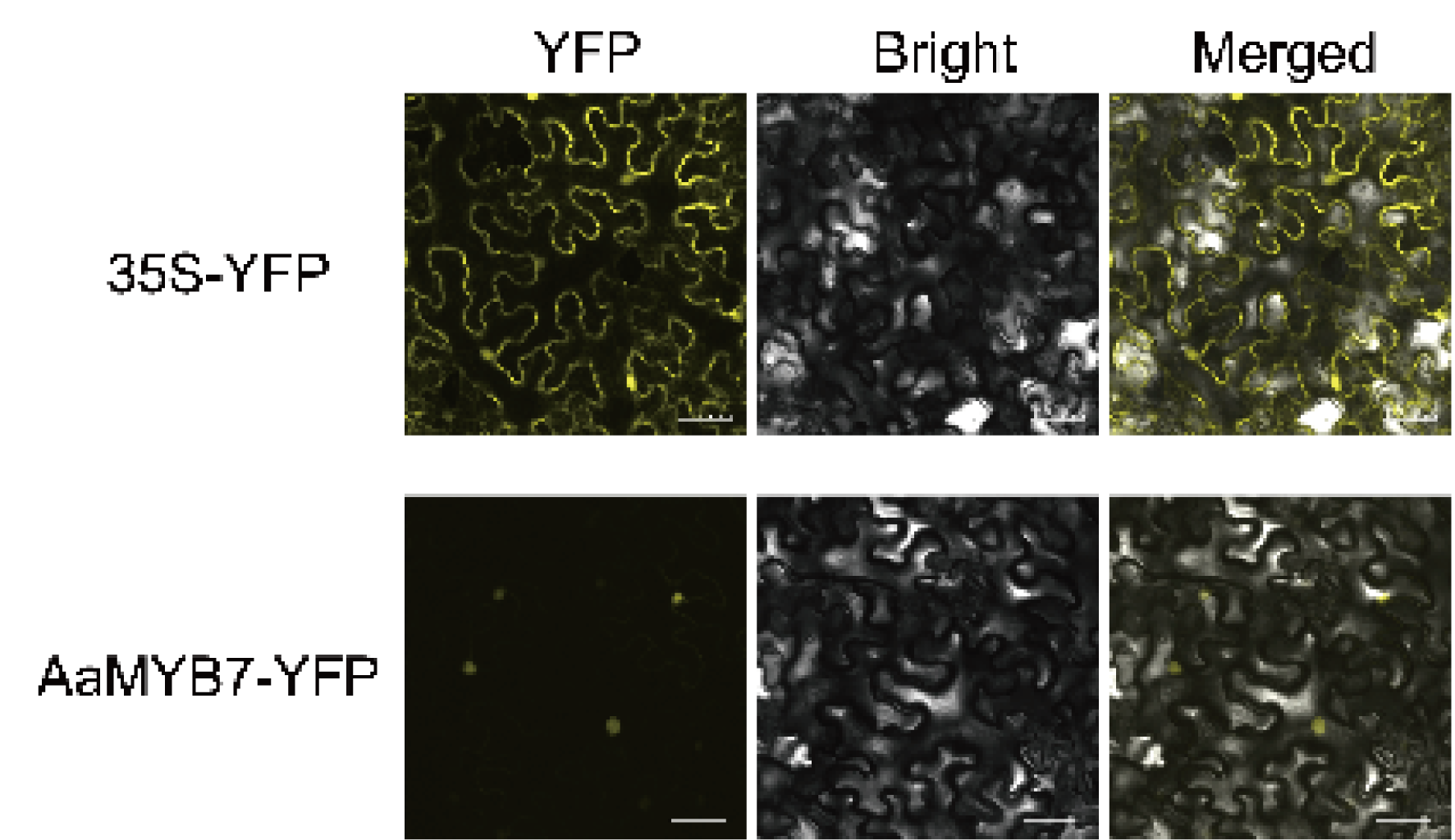

Supplement: Supplementary file 1 — Figure S1: Phylogenetic analysis and alignment of the protein sequences of AabHLH93 and related proteins. Figure S2: Subcellular localization of AabHLH93. Figure S3: Dual‐LUC assay showing that AaWRKY9 activates the expression of AabHLH93. Figure S4: Phylogenetic analysis and alignment of the protein sequences of AaMYB7 and related proteins. Figure S5: Subcellular localization of AaMYB7. Figure S6: Endogenous jasmonate levels and biosynthetic gene expression dynamics across the leaves at different developmental stages in Artemisia annua . Figure S7: The genetic transformation and PCR identification of AabHLH93 transgenic Artemisia annua lines. Figure S8: HPLC chromatograms of artemisinin. [file PBI-24-1533-s002.zip › pbi70416-sup-0001-FiguresS1-S8/pbi70416-sup-0001-FiguresS1-S8/pbi70416-sup-0006-FigureS5.tif]

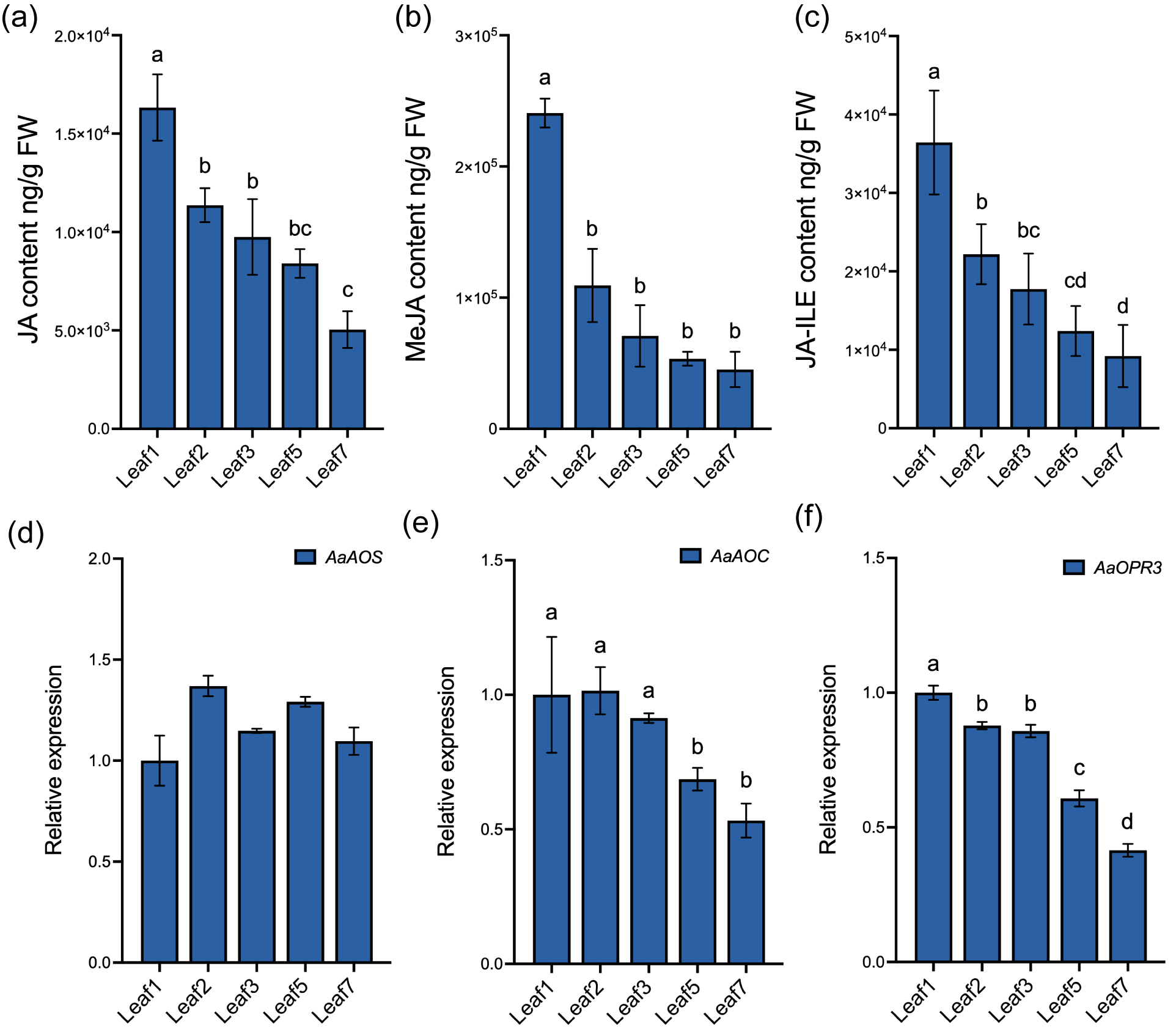

Supplement: Supplementary file 1 — Figure S1: Phylogenetic analysis and alignment of the protein sequences of AabHLH93 and related proteins. Figure S2: Subcellular localization of AabHLH93. Figure S3: Dual‐LUC assay showing that AaWRKY9 activates the expression of AabHLH93. Figure S4: Phylogenetic analysis and alignment of the protein sequences of AaMYB7 and related proteins. Figure S5: Subcellular localization of AaMYB7. Figure S6: Endogenous jasmonate levels and biosynthetic gene expression dynamics across the leaves at different developmental stages in Artemisia annua . Figure S7: The genetic transformation and PCR identification of AabHLH93 transgenic Artemisia annua lines. Figure S8: HPLC chromatograms of artemisinin. [file PBI-24-1533-s002.zip › pbi70416-sup-0001-FiguresS1-S8/pbi70416-sup-0001-FiguresS1-S8/pbi70416-sup-0007-FigureS6.tif]

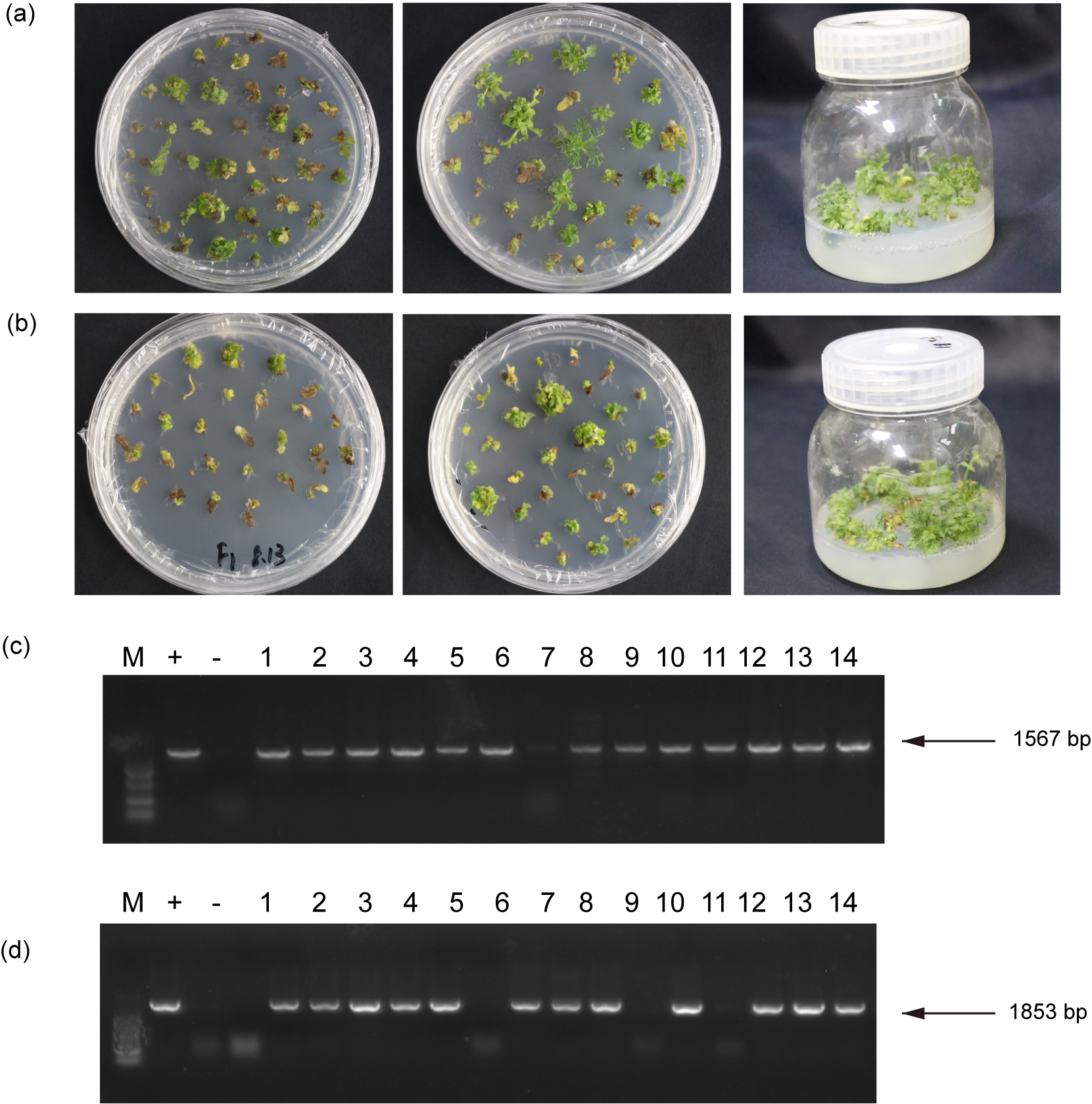

Supplement: Supplementary file 1 — Figure S1: Phylogenetic analysis and alignment of the protein sequences of AabHLH93 and related proteins. Figure S2: Subcellular localization of AabHLH93. Figure S3: Dual‐LUC assay showing that AaWRKY9 activates the expression of AabHLH93. Figure S4: Phylogenetic analysis and alignment of the protein sequences of AaMYB7 and related proteins. Figure S5: Subcellular localization of AaMYB7. Figure S6: Endogenous jasmonate levels and biosynthetic gene expression dynamics across the leaves at different developmental stages in Artemisia annua . Figure S7: The genetic transformation and PCR identification of AabHLH93 transgenic Artemisia annua lines. Figure S8: HPLC chromatograms of artemisinin. [file PBI-24-1533-s002.zip › pbi70416-sup-0001-FiguresS1-S8/pbi70416-sup-0001-FiguresS1-S8/pbi70416-sup-0008-FigureS7.tif]

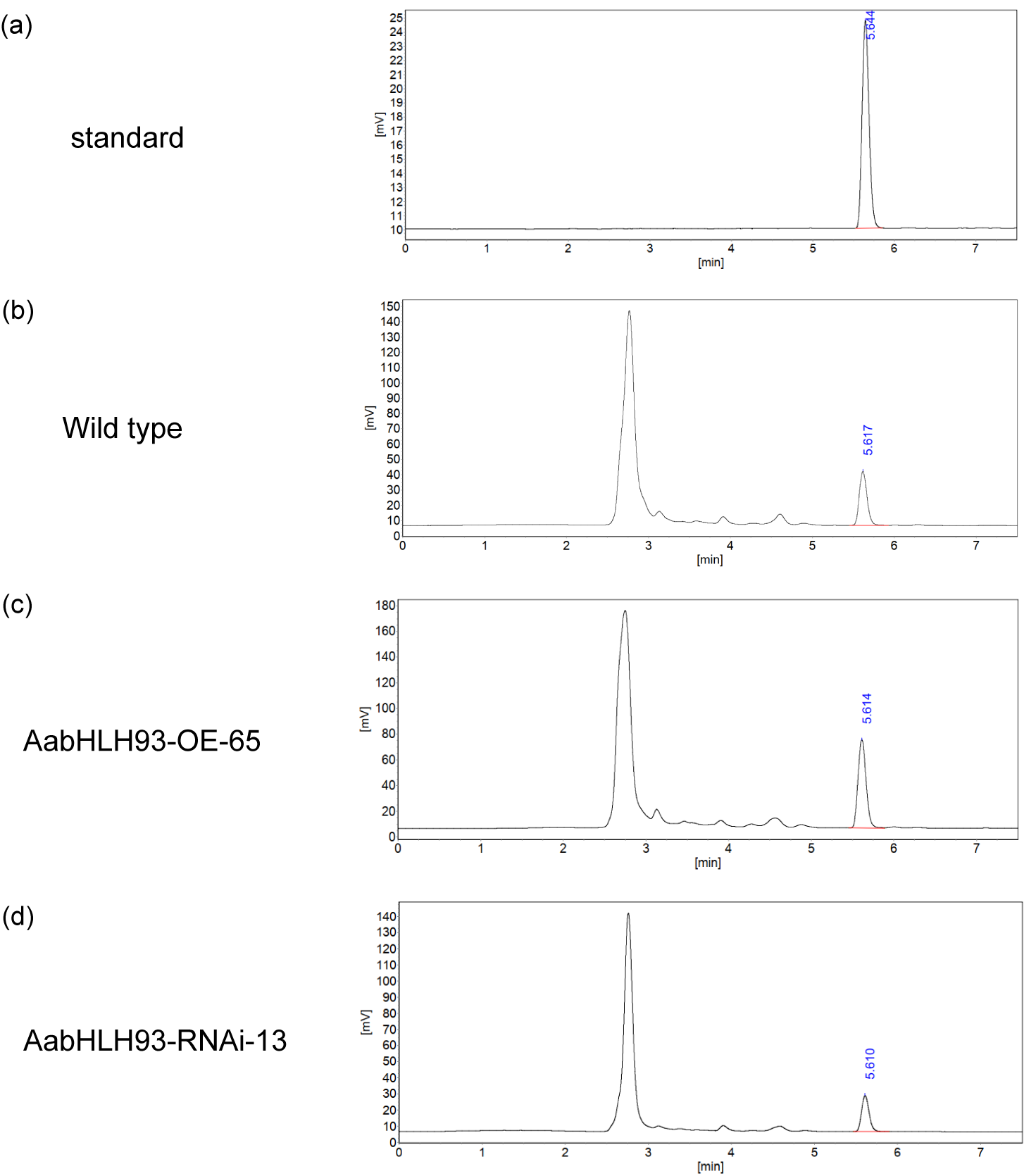

Supplement: Supplementary file 1 — Figure S1: Phylogenetic analysis and alignment of the protein sequences of AabHLH93 and related proteins. Figure S2: Subcellular localization of AabHLH93. Figure S3: Dual‐LUC assay showing that AaWRKY9 activates the expression of AabHLH93. Figure S4: Phylogenetic analysis and alignment of the protein sequences of AaMYB7 and related proteins. Figure S5: Subcellular localization of AaMYB7. Figure S6: Endogenous jasmonate levels and biosynthetic gene expression dynamics across the leaves at different developmental stages in Artemisia annua . Figure S7: The genetic transformation and PCR identification of AabHLH93 transgenic Artemisia annua lines. Figure S8: HPLC chromatograms of artemisinin. [file PBI-24-1533-s002.zip › pbi70416-sup-0001-FiguresS1-S8/pbi70416-sup-0001-FiguresS1-S8/pbi70416-sup-0009-FigureS8.tif]
